# Photoacoustic-Integrated Multimodal Approach for Colorectal Cancer Diagnosis

**DOI:** 10.1021/acsbiomaterials.5c00918

**Published:** 2025-07-01

**Authors:** Shimul Biswas, Diya Pratish Chohan, Mrunmayee Wankhede, Jackson Rodrigues, Ganesh Bhat, Stanley Mathew, Krishna Kishore Mahato

**Affiliations:** † Department of Biophysics, 539679 Manipal School of Life Sciences, Manipal Academy of Higher Education, Manipal, Karnataka 576104, India; ‡ Department of Life Science Informatics, b-it, Rheinische Friedrich-Wilhelms-Universität Bonn, Bonn 53113, Germany; § Institute of Biophotonics, National Yang Ming Chiao Tung University, Taipei 112304, Taiwan; ∥ Department of Gastroenterology and Hepatology, 29224 Kasturba Medical College, Manipal Academy of Higher Education, Manipal, Karnataka 576104, India; ⊥ Department of General Surgery, Kasturba Medical College, Manipal, Manipal Academy of Higher Education, Manipal, Karnataka 576104, India

**Keywords:** photoacoustic, colorectal, cancer, multimodal, imaging

## Abstract

Colorectal cancer remains a major global health challenge, emphasizing the need for advanced diagnostic tools that enable early and accurate detection. Photoacoustic (PA) spectroscopy, a hybrid technique combining optical absorption with acoustic resolution, is emerging as a powerful tool in cancer diagnostics. It detects biochemical changes in biomolecules within the tumor microenvironment, aiding early identification of malignancies. Integration with modalities, such as ultrasound (US), photoacoustic microscopy (PAM), and nanoparticle-enhanced imaging, enables detailed mapping of tissue structure, vascularity, and molecular markers. When combined with endoscopy and machine learning (ML) for data analysis, PA technology offers real-time, minimally invasive, and highly accurate detection of colorectal tumors. This approach supports tumor classification, therapy monitoring, and detecting features like hypoxia and tumor-associated bacteria. Recent studies integrating machine learning with PA imaging have demonstrated high diagnostic accuracy, achieving area under the curve (AUC) values up to 0.96 and classification accuracies exceeding 89%, highlighting its potential for precise, noninvasive colorectal cancer detection. Continued advancements in nanoparticle design, molecular targeting, and ML analytics position PA as a key tool for personalized colorectal cancer management.

## Graphical abstract

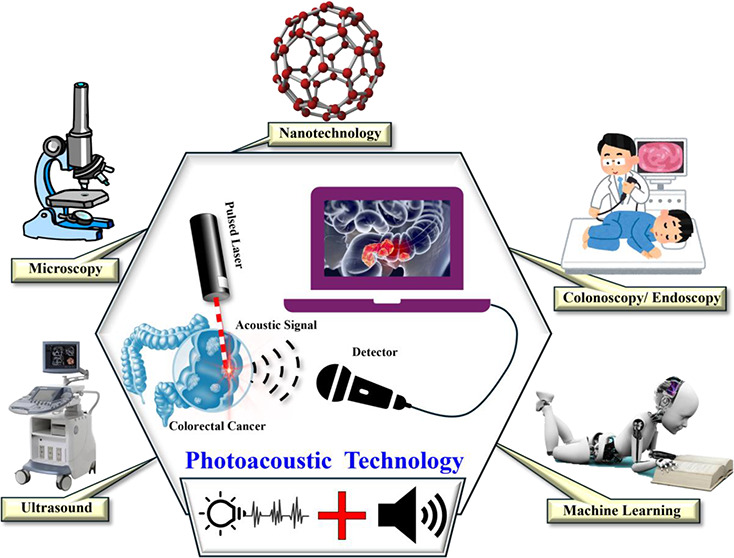

## 1 Introduction

Colorectal cancer (CRC) is a major global health issue, ranking as the third most common cancer all over the globe with an estimated 19,26,425 new cases. It is also responsible for the second-highest number of deaths related to the disease, causing 9,04,019 fatalities according to GLOBACON 2022. Over 50% of colorectal cancer cases occur in Asia, highlighting the region’s substantial burden of this disease (Figure 1).^1^ These statistics underscore the critical need for enhanced screening, early detection, and effective treatment strategies.^2^ The World Health Organization anticipates that by 2030, 75 million individuals will experience CRC, with 17 million fatalities and 27 million new diagnoses projected.^3^ Recent analyses reported 154,270 new cases and 52,900 deaths from colorectal cancer in the United States in 2025.^4^ Globally, recent peer-reviewed research confirms that colorectal cancer remains the third most common cancer, with more than 1.9 million new cases and almost 900,000 deaths annually, and underscores the concerning rise in incidence among individuals under 50 years old.^5^ These trends highlight ongoing challenges in colorectal cancer prevention, early detection, and management.

**Figure 1:**
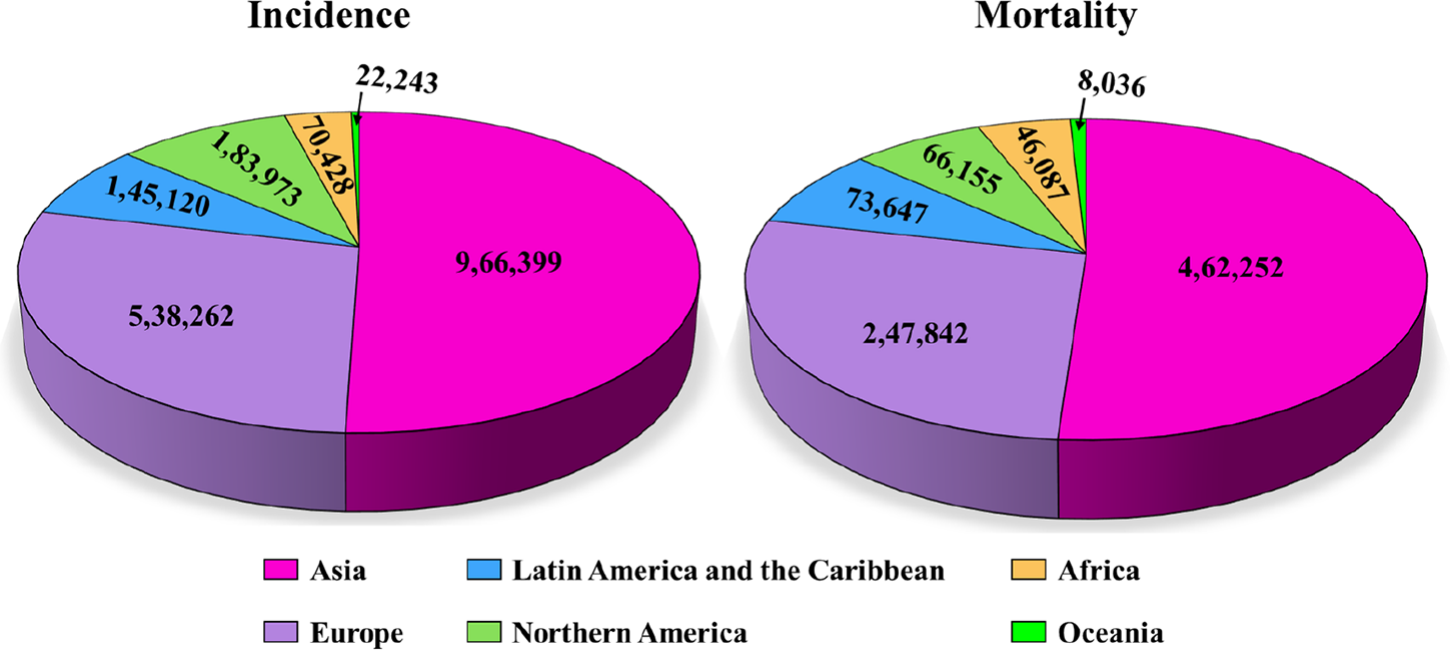
Incidence and mortality data of colorectal cancer all over the world by GLOBACON 2022.^1^

Understanding the progression of colorectal cancer is vital as it progresses through distinct stages, starting from stage 0, where abnormal cells are confined to the mucosa. Stage I shows growth into the muscular layer, while stage II involves deeper tissue penetration without lymph node involvement. Stage III indicates the spread to nearby lymph nodes, and stage IV, or metastatic cancer, marks the spread to distant organs like the liver and lungs, representing an advanced and more complex phase of the disease.^6,7^ Colorectal cancer develops through two main precursor pathways: the adenoma-carcinoma pathway (70–90% of cases) and the serrated neoplasia pathway (10–20%).^8,9^ In the adenoma-carcinoma pathway, APC mutations trigger chromosomal instability, leading to tumor formation, often followed by RAS activation or TP53 loss.^10^ The serrated pathway, on the other hand, is driven by RAS and RAF mutations and characterized by epigenetic changes, particularly CpG island methylation.^11^ This pathway can result in either microsatellite stable or unstable tumors. Additional mutations, such as those in POLE or mismatch repair genes, contribute to a hypermutated phenotype.^12^ Both pathways involve complex genetic and epigenetic alterations, with the adenoma-carcinoma pathway progressing through mutations, while the serrated pathway typically begins with BRAF or KRAS mutations, followed by gene methylation.^8,13,14^ Complementing these molecular insights, cytological evaluation techniques have recently advanced, enabling the detection of circulating tumor cells that reflect tumor heterogeneity and aid in monitoring disease progression.^15^

Early detection of CRC is important for reducing mortality and improving patient outcomes, as CRC is a leading cause of cancer-related deaths. Its nonspecific symptoms often lead to misdiagnosis, making effective screening essential for identifying the disease at earlier stages.^16^ While screening programs can improve prognosis and enable preventative measures like polypectomy, challenges such as technological limitations and patient barriers persist.^17^ Meanwhile, ongoing cancer biology research continues to identify novel molecular targets and therapeutic strategies, which hold potential to enhance early detection and enable more personalized treatment approaches.^18^ Pharmacological advances further complement these efforts by developing innovative drug delivery systems and adjuvant therapies designed to overcome resistance and improve patient outcomes in CRC.^19^

Addressing these issues is crucial for enhancing early diagnosis and reducing the financial burden of advanced-stage treatment, emphasizing the need for prioritizing early detection strategies for CRC.^20–22^ Several conventional diagnostic methods are currently used for colorectal cancer detection, each with distinct strengths and limitations (Table 1). Colonoscopy remains the gold standard for visualizing the entire colon and collecting biopsies. However, it lacks molecular specificity and requires invasive preparation.^23,24^ Histopathological analysis provides detailed cellular insight but is limited to sampled regions and is time-consuming.^25,26^ Noninvasive tests like the fecal immunochemical test (FIT) and stool DNA testing offer convenience but suffer from limited sensitivity and specificity, especially in early stage detection.^27–30^ Imaging techniques such as computed tomographic colonography,^31^ MRI,^32^ and capsule endoscopy^33,34^ provide anatomical visualization but are often limited by resolution, coverage, or inability to characterize tissue composition. Optical coherence tomography (OCT) delivers high-resolution, real-time imaging of tissue microstructure but is restricted by its shallow penetration depth and difficulty in distinguishing benign from malignant changes at early stages,^35,36^ while fluorescence imaging offers high molecular specificity, but is similarly limited by shallow penetration and often requires exogenous contrast agents for optimal results.^37–39^ In contrast, PA imaging offers a unique combination of high-resolution anatomical and molecular imaging capabilities, enabling noninvasive visualization of vascular and tissue-specific contrast without ionizing radiation. These features position PAI as a highly promising modality for improving the early diagnosis and characterization of colorectal cancer.^40^

**Table 1:** Comparison of PA Imaging with Conventional Technologies for Colorectal Cancer Detection, with Their Advantages and Limitations

technology	advantages	limitations	references
colonoscopy	• allows visualization of the entire colon and rectum	• requires bowel preparation	^23,24^
	• allows for the collection of tissue samples for histological examination	• it can only provide an image view, but it is incapable of distinguishing between adenoma and carcinoma	
biopsy and histopathology	• enables detailed analysis of cell types and structures, confirming malignancy	• limited to the area sampled	^25,26^
	• helps in determining the stage and grade of the cancer	• some lesions may be challenging to biopsy, especially if they are deep within the colon	
		• time-consuming for the results	
fecal immunochemical test (FIT)	• does not require insertion of instruments into the body	• detected blood may not always be indicative of cancer (false positives)	^27,28^
	• specifically detects human hemoglobin, making it more accurate for detecting lower gastrointestinal bleeding	• may miss some early stage cancers or small polyps (false negatives)	
stool DNA test	• noninvasive, requires only a stool sample	• a positive result necessitates follow-up with a colonoscopy for confirmation and further investigation	^29,30^
	• detects both DNA mutations associated with colorectal cancer and hidden blood in the stool	• false positives compared to FIT, leading to unnecessary follow-up procedures	
computed tomographic colonography	• less invasive than traditional colonoscopy and no need for sedation	• requires thorough bowel cleansing, which can be uncomfortable	^31^
	• provides detailed images of the colon and rectum, as well as surrounding tissues	• less effective at detecting very small polyps compared to traditional colonoscopy	
flexible sigmoidoscopy	• only examines the lower part of the colon and rectum, making it less invasive than a full colonoscopy	• only examines the lower third of the colon, potentially missing polyps or cancers in the upper colon	^41,42^
	• generally, less expensive than a colonoscopy	• abnormal findings typically require a follow-up colonoscopy for a complete examination	
magnetic resonance imaging	• provides high-resolution images of soft tissues, offering detailed views of the rectum and surrounding areas	• MRI is more commonly used for rectal cancer rather than the entire colon due to its specific imaging capabilities	^32^
capsule endoscopy	• involves swallowing a small, pill-sized camera, avoiding the discomfort associated with traditional endoscopy	• the capsule cannot be controlled once swallowed, which may result in missed areas or incomplete examination	^33,34^
	• patients do not need sedation, reducing risks and recovery time associated with anesthesia	• may not always visualize the entire colon due to variations in transit time or battery life	
optical coherence tomography	• high resolution (cellular scale, 4–20 μm)	• limited penetration depth (1–3 mm)	^35,36^
	• noninvasive, real-time imaging	• difficulty distinguishing benign inflammation from early cancer	
	• good for early detection and guided biopsy	• narrow field of view	
	• can visualize subsurface microvasculature	• operator-dependent	
	• superior to white light endoscopy in sensitivity and specificity for dysplasia and cancer	• high cost and specialized training required	
		• may miss larger or diffuse lesions	
fluorescence imaging	• high molecular specificity	• shallow tissue penetration (typically <1 mm)	^37–39^
	• real-time visualization of targeted biomarkers	• Requires exogenous contrast agents	
	• sensitive to early molecular changes	• background autofluorescence can reduce specificity	
	• useful for intraoperative guidance and cell tracking	• limited to surface or superficial lesions	
	• reduces anastomotic leakage and improves lymph node detection in surgery	• not widely standardized for CRC	
photoacoustic imaging	• high optical absorption contrast (detects endogenous chromophores like hemoglobin, melanin, and exogenous nanoparticles)	• limited clinical adoption and standardization	^40,43,44^
	• deep tissue penetration (up to 4–5 cm depending on tissue type)	• image quality and resolution depend on tissue optical properties and depth	
	• nonionizing, noninvasive, and real-time imaging	• requires robust laser and ultrasound systems	
	• combines anatomical (US) and functional (optical) information	• can be affected by motion artifacts in vivo	
	• capable of molecular and cellular characterization with targeted contrast agents	• may require exogenous contrast for specific molecular targets	
	• enables visualization of tumor angiogenesis and vascular abnormalities		
	• sensitive to early changes in tumor vasculature and hypoxia		
	• can be integrated with machine learning for automated analysis and improved diagnostic accuracy		

Emerging approaches, such as the integration of traditional medicine and modern diagnostic modalities, are being explored to enhance the sensitivity and specificity of CRC screening.^45^ Recent developments in nanotechnology have enabled the design of multifunctional nanoparticles for targeted imaging and therapy, offering new possibilities for noninvasive CRC diagnosis.^46^ The application of advanced nanomaterials in photoacoustic imaging has shown significant potential to enhance the detection and characterization of CRC lesions, showing the way for more precise and individualized diagnostic strategies.^47^ Recent studies have also highlighted the growing importance of artificial intelligence and deep learning approaches in predicting CRC outcomes and stratifying patient risk, which may help address some of these ongoing challenges.^48^

Photoacoustic spectroscopy is a method used to detect biomolecular signals by taking advantage of the photoacoustic effect. When a sample is stimulated with a specific wavelength of modulated or pulsed light, the molecules in the sample absorb the light and become excited, moving to higher energy states. Due to nonradiative relaxation, this leads to localized heating, followed by expansion and relaxation, creating pressure variations that appear in the acoustic region of the frequency domain, known as photon-induced acoustic signals or photoacoustic signals. These acoustic signals can be detected using suitable acoustic detectors like microphones or PZT, and analyzing the waves reveals the optical absorption characteristics of the biomolecules of a sample under investigation.^49–51^

When combined with complementary imaging technologies such as ultrasound, photoacoustic microscopy, and nanoparticle-enhanced imaging, PAI offers a comprehensive view of CRC tumors, providing not just anatomical but also functional and molecular data. This integration allows for the visualization of vital tumor features such as vascularity, oxygenation, and biomarker expression, leading to more accurate diagnoses and monitoring of CRC.^44,52,53^ The incorporation of ML into PAI enhances the processing and interpretation of imaging data, enabling faster, more precise identification of key biomarkers that can guide individualized treatment plans.^54^

As PAI technology advances, its potential to transform CRC diagnosis and management grows. By offering real-time, noninvasive imaging and enhanced monitoring capabilities, PAI could revolutionize how CRC is diagnosed and treated.^44^ Although challenges such as regulatory approval, cost, and molecular targeting need to be addressed, the future of PAI in CRC care holds great promise. This article delves into the current state of PAI, its integration with other diagnostic technologies, and the path forward for overcoming existing obstacles and fully realizing its clinical potential.

## 2 Photoacoustic Integrated Multimodal Approach for Colorectal Cancer Detection and Diagnosis

### 2.1 Photoacoustic Ultrasound

Photoacoustic imaging is a hybrid modality that merges the high optical contrast of light-based imaging with the deep tissue penetration capability of ultrasound. In this method, a pulse laser light is illuminated at the tissue, where light-absorbing chromophores such as hemoglobin, collagen, certain lipids, etc., absorb the energy. This absorbed light generates heat, causing rapid thermoelastic expansion followed by contraction due to the pulsed/modulated nature of light excitation to the sample, generating acoustic waves. Ultrasound transducers detect these waves, which are then processed to create high-resolution images.^55,56^ By combining light and sound, photoacoustic imaging utilizes the benefits of optical contrast and ultrasound resolution, making it a powerful tool for applications such as cancer diagnosis, vascular imaging, and tissue characterization.^51,57,58^ The versatility and clinical promise of photoacoustic tomography (PAT) have been demonstrated across a range of studies, showing its capability for noninvasive, high-resolution imaging from the organelle level to entire organs. Foundational work has established practical guidelines for PAT in the life sciences and highlighted its use in multimodal functional imaging of the intestine, supporting its translation into gastrointestinal and colorectal cancer diagnostics.^59–61^

Yang et al. used a coregistered photoacoustic tomography (PAT) and US system to image human colorectal tissues ex vivo, integrating a Q-switched Nd:YAG laser for light delivery. The PAT system delivered pulsed laser light at a wavelength of 750 nm, with a pulse duration of 10 ns and a repetition rate of 15 Hz. Imaging was performed across four wavelengths of 730, 780, 800, and 830 nm with an overall scanned region measured between 1 and 3 cm in size, achieving a frame rate of 15 frames per second. Data analysis included calculation of relative oxy- and deoxy-hemoglobin (rHbO_2_ and rHb) to assess tissue oxygenation, spectral features like spectral slope and intercept, and textural properties derived from gray-level co-occurrence matrices (GLCM). US images identified regions of interest for PAT analysis, while PAT features were further processed using Gaussian fits and Radon transforms. The study demonstrated clear distinctions between malignant and normal tissues based on tissue architecture and spectral parameters, highlighting the potential of PAT/US for enhanced colorectal cancer diagnosis and monitoring.^44^ Another study demonstrates the feasibility of using an LED-based PAI system, AcousticX, for *ex vivo* colon cancer tissue analysis. The system combines US and PAI for coregistered imaging, utilizing 850 nm LEDs instead of lasers. The experimental setup includes a motorized stage for precise probe movement and a custom-designed water container with a polyurethane film for impedance matching. The LED-based probe scanned a 20 mm section of a fresh colon cancer specimen. Analysis revealed that malignant tissues exhibit weaker PA signals than healthy and fatty tissues, with signal variability observed between different regions. A key advantage of this setup is its portability and semiautomated data acquisition.^62^ The combination of the PA and US technology for colorectal cancer diagnosis was also explored by Ikematsu et al., who developed a PA/US echo imaging system integrating a linear ultrasound transducer array with a pulsed Alexandrite laser (750 nm wavelength, 50 ± 10 ns pulse width, 10 Hz repetition rate) to excite photoacoustic waves. The system achieves real-time coregistration of B-mode US and PA images, enabling precise visualization of blood vessel networks in gastrointestinal (GI) tissue layers. The scanning process captures high-resolution 2D and 3D images with a spatial resolution of 0.3 mm, detecting microvessels as small as 30 μm at depths up to 20 mm (Figure 2). The PA/US combination enhances colorectal cancer detection by visualizing vascular changes, such as thickened and prominent vessels in submucosal invasive cancers, which are indicative of malignancy. This dual-modality approach improves diagnostic accuracy, providing insights into cancer invasion depth and aiding in treatment planning.^63^

**Figure 2:**
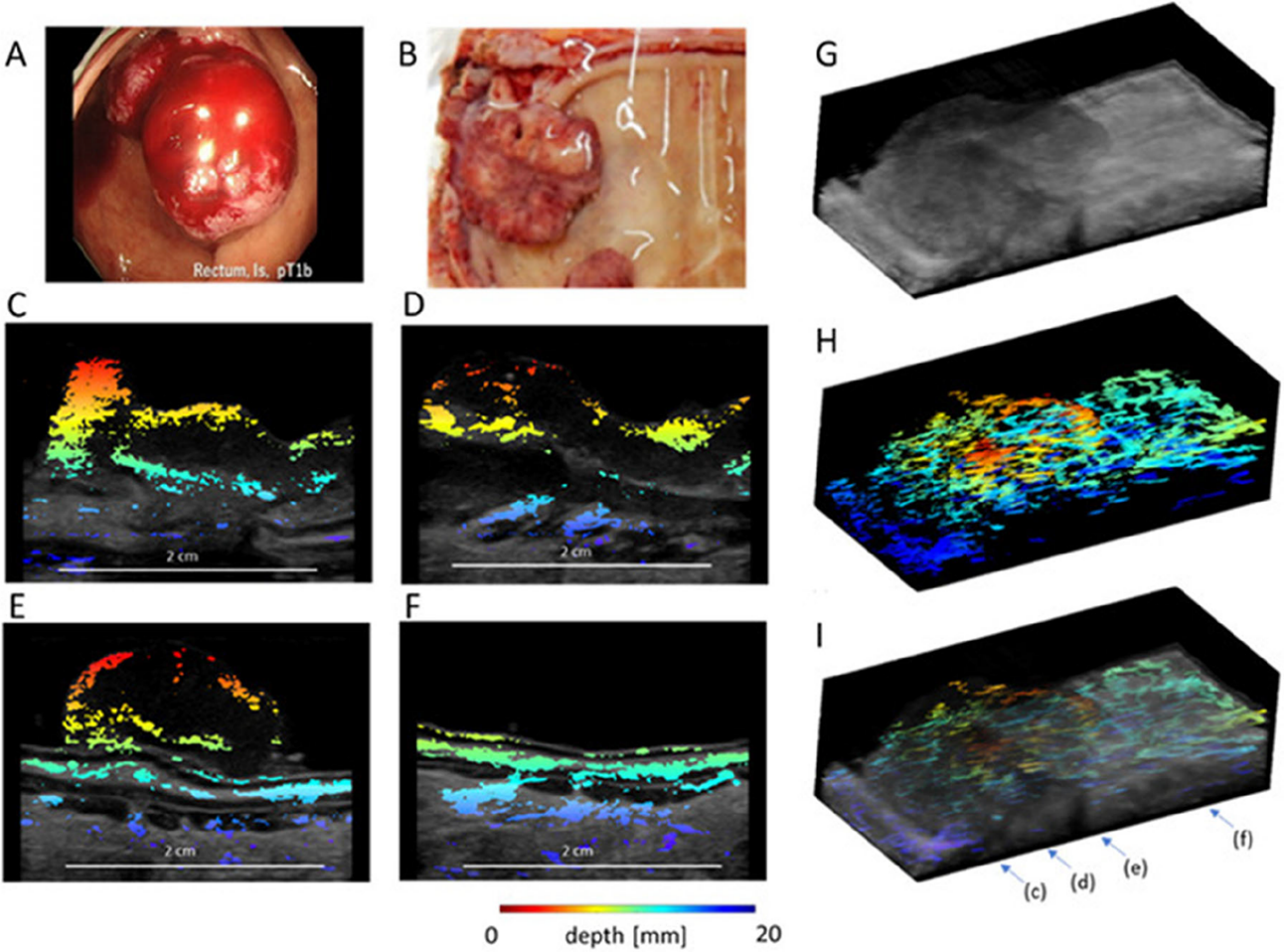
Multimodal imaging of a rectal T1 lesion using a combined 3D PA and US imaging system. (A) Endoscopic view of the rectal T1 cancer and (B) resected specimen prepared for imaging. (C–F) Cross-sectional 2D PA images superimposed on grayscale US images, revealing detailed vascular structures and tissue layers with depth color coding. (G) 3D US image of the specimen, providing structural insights and (H) 3D PA image, highlighting vascular networks at different depths. (I) Integrates the 3D PA and US images, enabling coregistered visualization of structural and vascular details. The arrows in (I) indicate the positions of the cross sections shown in (C–F)*,* and the color bar represents depth from the probe surface, with red indicating shallow regions and blue representing deeper areas. (Reproduced from ref 63. Available under a CC-BY 4.0 license. Copyright 2021 Wiley.)

PAT has significantly advanced CRC detection by integrating photoacoustic imaging with innovative contrast agents like cyanine and hemicyanine dyes. These dyes provide tunable tumor-targeting capabilities, distinct near-infrared (NIR) absorption at 700–800 nm, and exceptional stability in biological environments. The Cy-HCy-H2S probes stand out by offering precise ratiometric responses to hydrogen sulfide (H_2_S), a key CRC biomarker, greatly enhancing detection specificity. These probes target tumor mitochondria by switching between “caged” and “uncaged” states. With 808 nm laser excitation and high-resolution detectors, PAT systems are achieving precise localization of CRC tumors. Overall, these advancements enable noninvasive, highly specific imaging that transforms early diagnosis and surgical intervention.^64^ Lin et al. used an in-house coregistered US-PA imaging system with a side-viewing endorectal probe. This approach combines structural information from the US and functional data from PA imaging as an integrated technique to assess rectal cancer treatment response. The probe features a 20 MHz transducer and a 1064 nm Nd:YAG laser, delivering high-resolution imaging. This dual-modality system captures 360° coregistered B scans with an imaging depth of 24.6 mm. By exploring DenseNet architecture for classification, the system processes coregistered US-PA regions of interest (ROIs) alongside individualized normal tissue references, achieving improved diagnostic accuracy of 92.4%. Functional imaging through PA highlights disrupted cancer vasculature, complementing structural US data and enabling precise differentiation between cancerous tissue, scar regions, and treatment-responsive areas (Figure 3). This combined approach improves the specificity and clinical utility of imaging-guided colorectal cancer diagnosis.^65^ The integration of ultrasound with photoacoustic tomographic imaging has enabled significant advancements in colorectal cancer detection. Future work should aim to expand the study to include a larger and more diverse set of specimens, refine and optimize analysis methodologies for improved accuracy, and incorporate reference objects to enable more quantitative, depth-sensitive, and clinically translatable assessments.^66^

**Figure 3:**
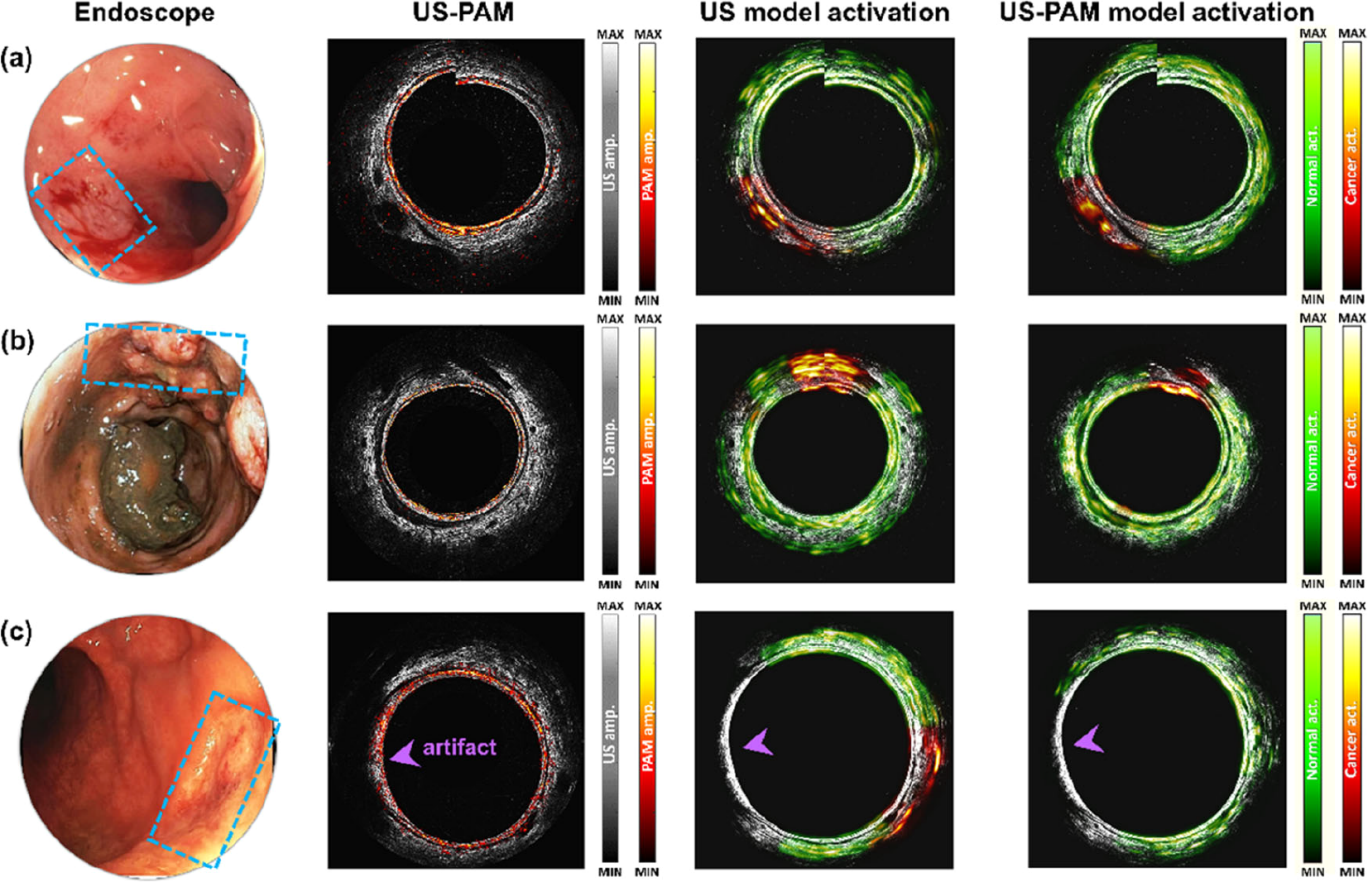
Representative images from patients in the testing cohort with US-PAM DenseNet predictions. Endoscopic photos with blue boxes highlight cancer sites. The US model activation and US-PAM activation columns display attention heat maps, where green indicates normal tissue and red represents cancer regions. (a) T3 cancer patient with minimal treatment response. (b) T2 cancer patient showing extensive scarring. (c) Complete treatment responder with no residual cancer. A purple arrowhead indicates an artifact due to poor ultrasound signal coupling, correctly classified by both the US model and US-PAM DenseNet. (Reproduced from ref 65. Available under an Optica Open Access Publishing Agreement. Copyright 2023 Optica Publishing Group.)

Despite these advantages, the combined PA/US modality faces challenges, including limited penetration depth of PA signals in highly scattering tissues, which can restrict imaging of deep-seated lesions. Additionally, the complexity and cost of integrated systems may hinder their widespread clinical adoption. Operator dependency and the need for specialized training further limit consistent image acquisition and interpretation.^67–69^ Addressing these limitations is essential to fully realize the clinical potential of PA/US imaging in colorectal cancer diagnosis.

### 2.2 Photoacoustic Microscopy

Photoacoustic microscopy (PAM) is an in vivo imaging technique that combines optical excitation and acoustic detection for high-resolution images based on optical absorption contrast. It can image depths of several millimeters and visualize a broad range of molecules. PAM is effective for providing simultaneous anatomical, functional, and metabolic information, highlighting its potential in biomedical research and clinical applications.^70,71^ Mechanistically, PAM uses pulsed laser light to excite tissue chromophores such as hemoglobin, which absorb the optical energy and generate localized heat through nonradiative relaxation. This heat causes thermoelastic expansion, producing ultrasonic waves detected by an acoustic transducer aligned with the optical focus. The amplitude of these waves correlates with the local optical absorption, enabling high-contrast imaging of tissue structures and functions.^72,73^ PAM operates in two modes: optical-resolution PAM (OR-PAM) for submicron lateral resolution by tightly focusing light, and acoustic-resolution PAM (AR-PAM) for deeper imaging using focused ultrasound detection with slightly lower resolution.^72,74,75^ This dual capability allows PAM to visualize microvasculature, oxygen saturation, and molecular composition at cellular to tissue scales, making it valuable for detailed colorectal cancer imaging.

Acoustic-resolution Photoacoustic Microscopy (AR-PAM) represents a significant advancement in the early detection of colorectal cancer by integrating high-resolution ultrasound and photoacoustic imaging within a compact endoscopic setup. The system uses a 750 nm laser delivered via a multimode fiber and a 20 MHz focused ring transducer for coregistered imaging. A 10 mJ laser pulse, focused onto a 0.6 cm^2^ tissue area with a safe fluence of 16.7 mJ/cm^2^, captures vascular architecture and tissue morphology with lateral and axial resolutions of 65 and 45 μm, respectively. The results obtained through the implementation of this technique revealed critical differences in vascularity and structural integrity between normal, benign, and malignant tissues, with malignant areas showing disrupted layering, hypovascular centers, and distinct spectral slopes in quantitative analysis. These features highlight AR-PAM’s ability to differentiate tissue types, detect early malignancies, and assess treatment responses, potentially revolutionizing colorectal cancer diagnosis and management.^76^ Another study published by Kou et al. has explored the optimization of PAM integrated with US imaging to enhance colorectal cancer detection by combining high-resolution imaging with vascular insights. The system described in the study utilizes a diode-pumped ND:YAG laser at 1064 nm, paired with advanced optics and a custom endoscopic probe for coregistered PAM and US imaging. Key features include vibration-damped mounting and synchronized acquisition with LabVIEW, achieving a frame rate of about 0.9 Hz. Optimal imaging parameters were established, with frequency ranges of 3–30 MHz for PAM and 9–22 MHz for US, enabling precise detection of colorectal abnormalities. Ex vivo and in vivo studies showed the system’s ability to differentiate normal, cancerous, and post-treatment tissues by analyzing perfusion and tissue structure. This PAM/US combination offers real-time, noninvasive diagnostics and monitoring for more personalized management of rectal cancer.^52^ The integration of high-resolution imaging and deep learning techniques has notably improved the detection of colorectal cancer through PAM combined with US. In a study involving 10 patients who completed preoperative chemoradiation therapy, in vivo imaging with a PAM/US endoscope was conducted before surgery, while *ex vivo* analyses were performed on specimens from 24 additional patients. The PAM endoscope, equipped with a 360° imaging head and laser-ultrasound capabilities, captured distinctive vascular and architectural disruptions associated with malignancy. A deep-learning model, particularly the PAM-CNN, outperformed traditional statistical classifiers with an AUC of 0.96 versus 0.82, accurately distinguishing normal from cancerous tissue (Figure 4). This innovation holds promise for improving the assessment of pathological responses in rectal cancer, potentially avoiding unnecessary surgeries for patients achieving complete tumor destruction post-treatment while reducing healthcare costs and improving outcomes.^77^ However, PAM is limited by shallow imaging depth due to optical scattering, the need for acoustic coupling media, and slower imaging speeds when high resolution is required, which can restrict its routine clinical use.^78,79^

**Figure 4:**
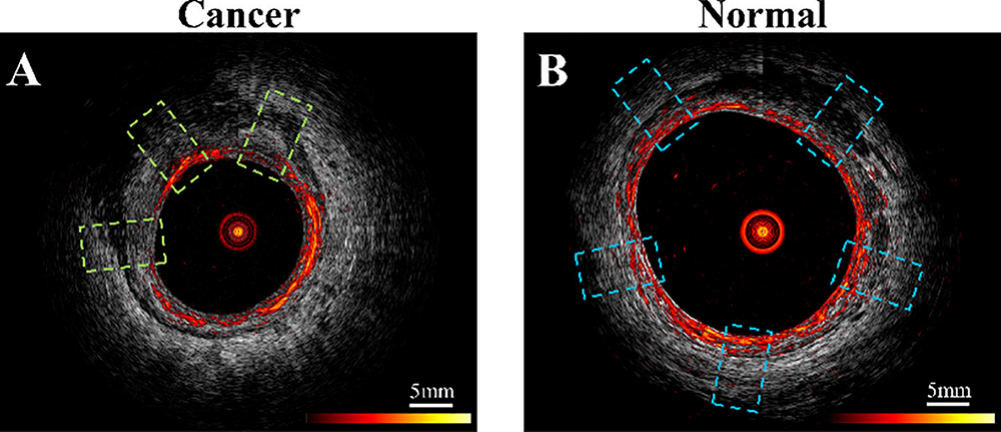
Co-registered photoacoustic microscopy (PAM) and ultrasound (US) images showcasing regions of interest (ROIs) for colorectal tissue assessment. (A) Residual cancer tissue highlighted with green dashed-line boxes and (B) normal tissue indicated with blue dashed-line boxes. ROIs are cropped independently from PAM and US images, emphasizing distinct tissue characteristics. (Reproduced from ref 77. Available under a CC-BY license. Copyright 2021, Frontiers.)

### 2.3 Photoacoustic Colonoscopy/Endoscopy

A colonoscope/endoscope is a long, flexible, tubular medical tool that is used to look within the GI tract. Generally, the length of the scope ranges from 160 to 180 cm, with a diameter between 1.0 and 1.2 cm, depending on the manufacturer and intended use. At the distal end of the colonoscope, several essential components facilitate its function. These include a camera for image and videography, and LEDs that ensure proper illumination of the colonic mucosa. Additionally, there is another channel to spray water or air into the colon to help clear debris, a camera lens, or facilitate visualization during the procedure. Another channel is used for inserting various instruments, such as biopsy forceps, for collecting tissue samples.^24,80^ Integrating PA imaging with colonoscopy or endoscopy involves delivering pulsed laser light through the endoscope to excite tissue chromophores, which generate ultrasonic waves via thermoelastic expansion. These photoacoustic signals are detected by miniaturized ultrasound transducers built into the endoscopic probe, enabling real-time, cross-sectional imaging of both superficial mucosa and deeper tissue layers. This approach provides functional and molecular contrast in addition to the structural information on conventional endoscopy, allowing improved visualization of vascular changes and tissue abnormalities in the gastrointestinal tract.^81–83^

Photoacoustic Imaging Endoscopy (PAIE) combines photoacoustic technology with endoscopy for noninvasive detection of internal lesions, particularly colorectal cancer. The PAIE system features a coaxial arrangement of light, sound, and a ring transducer array with 64 elements, allowing for 360° signal acquisition without transducer rotation. A Nd:YAG laser delivers pulsed energy through an optical fiber, creating photoacoustic signals that are processed by parallel acquisition algorithms. The system targets differences in the optical absorption coefficients of normal (0.043 mm^–1^) and cancerous tissue (0.163 mm^–1^) at 1065 nm. Using a limited-view filtered backprojection algorithm, the system produces high-contrast images with radial resolutions of about 0.32 mm and transverse resolutions of approximately 2.4 mm. The study showed the ability of the system to differentiate colorectal cancer tissue from normal tissue in *ex vivo* conditions, suggesting promise for future in vivo applications.^84^ Liu et al. introduced a dual-modality diagnostic system combining photoacoustic endoscopy (PAE) and hyperspectral imaging (HSI) for lower digestive tract diseases. The system features a miniature Q-switched laser for photoacoustic excitation, a polyvinylidene fluoride ultrasonic transducer for signal detection, and a liquid crystal tunable filter-based HSI system for optical imaging. PAE delivers structural imaging with 60 μm radial resolution and a depth of 2 mm, while HSI maps oxygen saturation and quantifies hemoglobin. Experiments in a rabbit rectum revealed critical vascular and oxygenation changes for identifying pathophysiological conditions. The image processing utilized spectral normalization and Lambert–Beer modeling to highlight key biomarkers like oxyhemoglobin and deoxyhemoglobin, enhancing subsurface imaging depth and functional analysis compared to conventional endoscopy.^85^ Another study showcases the integration of photoacoustic endoscopy (PAE), ultrasound endoscopy (USE), and wide-field optical microscopy for improved colorectal cancer imaging. The AR-PAE system uses a 532 nm pulsed laser, a parabolic mirror, and a 10 MHz ultrasound transducer, allowing for high-resolution imaging with centimeter-scale tissue penetration. The biomarker indocyanine green (ICG) enhances tumor contrast due to its absorption properties and deep tissue penetration. Image processing includes a modified back-projection algorithm for reconstructing layered structures and analyzing blood oxygen saturation and ICG diffusion. USE provides anatomical insights, while wide-field microscopy captures superficial vascular details. Overall, this system appears promising for colorectal cancer diagnostics.^86^ The multimodal imaging technique combining the high-resolution structural imaging of ultrasound with the functional and molecular sensitivity of photoacoustics to visualize vascular dynamics and tissue architecture in real time offers a cutting-edge approach to enhancing CRC detection. By highlighting hemoglobin-rich regions, photoacoustic imaging provides a detailed map of vascular perfusion, which is often disrupted in cancerous tissues. The endoscopic configuration allows in vivo imaging of internal colorectal regions during clinical procedures, as demonstrated in Figure 5, where the normal tissue exhibits intact vascular and layered structures, while the cancerous region shows disrupted perfusion and invasion into the serosa. This enables surgeons to distinguish between normal and malignant tissues with greater precision.^52^ The integration of PA technology with colonoscopy/endoscopy offers a significant advancement in colorectal cancer detection by enabling deeper tissue imaging with high-resolution insights. While studies using *ex-vivo* and animal models show promise, it is essential to translate this innovation for human use to confirm its clinical effectiveness. Future research should focus on developing flexible, patient-compatible systems and conducting human trials to validate its diagnostic capabilities. Despite promising advances, photoacoustic endoscopy faces challenges such as limited imaging depth due to optical scattering, the technical complexity of miniaturized probes, and the need for further validation in human clinical studies before routine adoption.^81,87,88^

**Figure 5:**
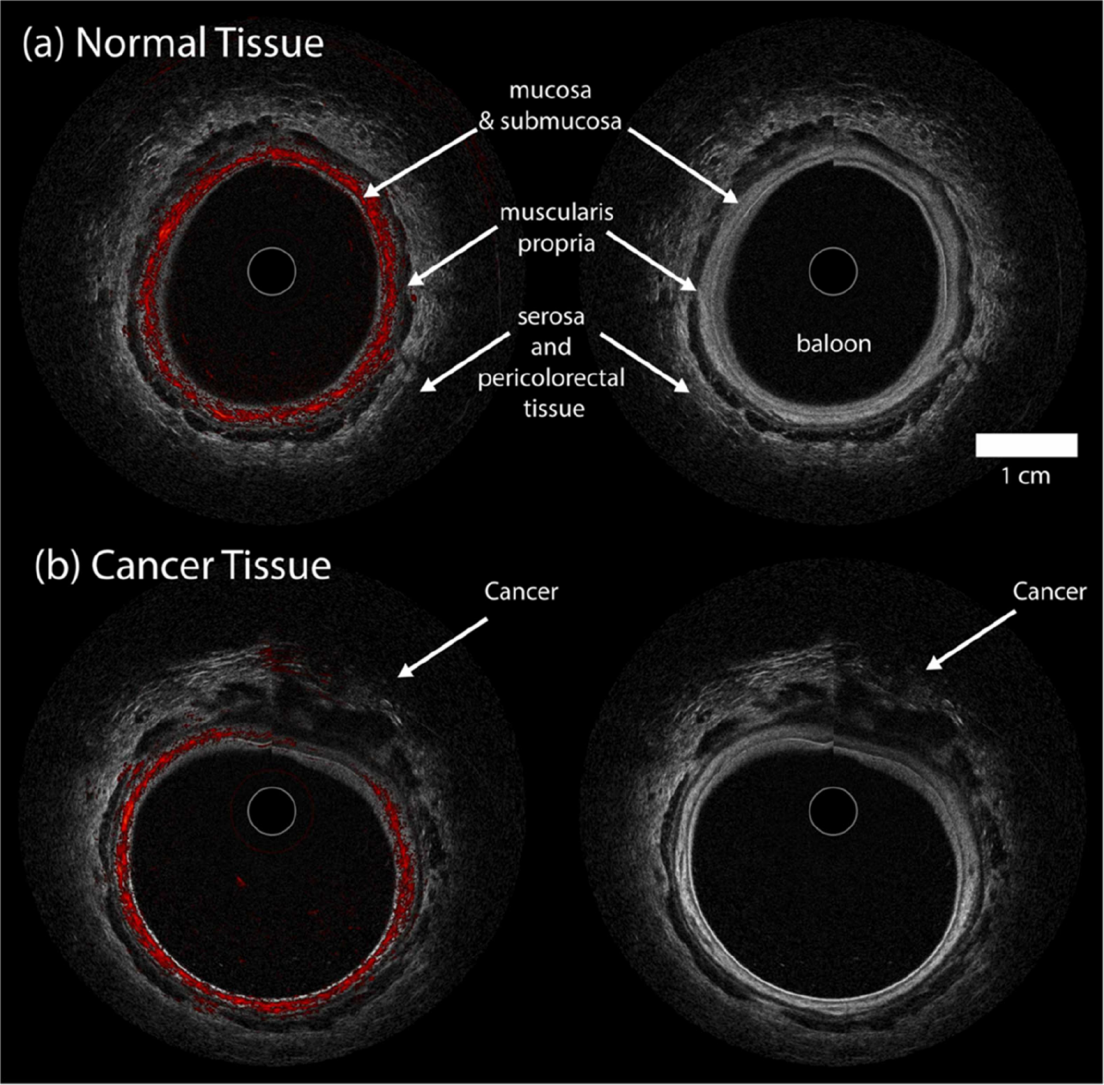
In vivo imaging of colorectal tissue: Coregistered ultrasound and photoacoustic images of (a) proximal normal region and (b) distal cancerous region in a 63-year-old male with invasive adenocarcinoma involving the muscularis propria. The PA signal highlights vascular features predominantly in the submucosa with minimal contributions from the muscularis propria and scattered vessels in the serosa. The cancerous region exhibits disrupted perfusion and structural invasion into the serosa, as indicated by the US image, showing loss of normal tissue architecture compared to the intact layered structure of normal tissue. (Reprinted with permission from ref 52. Available under a CC-BY-NC license. Copyright 2024, Elsevier.)

### 2.4 Photoacoustic Nanotechnology

Recent advances in nanotechnology have enabled the development of multifunctional nanoparticles for cancer imaging and therapy, offering enhanced contrast, targeted delivery, and integration with other modalities of imaging for improved diagnostic and therapeutic outcomes.^89–91^ Nanoparticles (NPs) are often used as contrast agents in techniques such as Photothermal therapy, Photothermal Imaging, PA Tomography, and PA Imaging. Nanoparticles can be used in photothermal therapy for colorectal cancer (Table 2) by serving as carriers for photothermal agents, enhancing tumor targeting, increasing stability, and improving the efficiency of localized heat generation upon light activation, thereby enabling precise and minimally invasive tumor ablation.^53,92,93^ These contrast agents have higher light absorption efficiency as compared to the endogenous chromophores present within the body. This ensures that the light absorbed by the site of interest is more ‘targeted’, which results in an enhanced PA signal. In addition, these external agents also help in increased optical energy to thermal energy conversion, resulting in the generation of stronger acoustic waves. These NPs can be highly modified according to need, and their functionalization in terms of shape, size, and composition can aid in the targeted imaging of specific tissues, for example, tumor tissues, which can help in obtaining an amplified signal from the regions of interest.^94^ For example, a study published by Liu et al. describes the synthesis of multifunctional NPs (metal–organic framework Fe NPs loaded with oxaliplatin and indocyanine green, modified using hyaluronic acid). These synthesized NPs showed strong optical absorption in the NIR region of the electromagnetic spectrum, showcasing their potential to be good PAI contrast agents. The PA imaging ability of the synthesized NPs was evaluated in an in vivo system, and a concentration-dependent increase in the PA signal was recorded. The study also described how PA imaging can be used for Imaging-guided Therapy and be synergistically combined with Chemo-PTT and Immunotherapy in the overall detection and treatment of colorectal cancer.^95^ The manifestation of enteropathogenic bacteria, (Fn), is seen to be significantly increased in CRC tissue as compared to healthy tissue, worsening the cancerous condition by promoting the metastasis of the tumor.^96^ The major problem associated with this abnormal proliferation of bacteria at the cancer site is the reduced efficiency of chemotherapeutic drugs due to bacterial-induced drug resistance.^97^ Xin et al. demonstrated the engineering of an in situ activated antitumor and antibacterial platform that can be used for the destruction of both (i) CRC cells and (ii) , improving the overall efficiency of anticancer therapies such as photothermal therapy (PTT). NIR-laser-triggered photothermal therapy is an emerging technique that uses different photothermal agents, which can be specifically concentrated at the site of the tumor. The NIR radiation can penetrate the soft tissues up to 2 cm, and the tumor-site concentrated photothermal agents absorb this incoming optical energy and convert it into thermal energy, resulting in the formation of a hyperthermic environment.^98^ This temperature rise (above 42 °C) results in the selective ablation of the cancer cells,^99^ and the damage generally caused to the surrounding normal cells via the bystander effect in other chemotherapeutic strategies can be avoided. In the study mentioned above, a smart Cu_2_O/BNN6@MSN-Dex nanoplatform was constructed, which was selectively accumulated at the tumor site. Overexpression of H_2_S at the CRC site results in the sulfurization of the Cu_2_O particles, leading to the formation of copper sulfide. This endogenously formed copper sulfide has excellent photothermal and photoacoustic properties. The PA signal obtained for the tumor imaging after administration of the nanoplatform was significantly higher than before. These imaging results are extremely useful for real-time monitoring of the nanoplatform.^100^ Nanoparticle-based photothermal ablation (PTA) using biocompatible agents like IR820@PEG-SPIO shows promising multimodal imaging and effective tumor ablation in deep cancers such as hepatocellular carcinoma. Minimally invasive laparoscopic-assisted PTA combines precise imaging-guided therapy with reduced toxicity and improved outcomes, offering potential clinical translation beyond surface tumors.^101^

**Table 2:** Most Commonly Used Nanoparticles in CRC-Specific PA Imaging

type of nanoparticle	key features for PA imaging	examples/applications in CRC
gold nanoparticles	high optical absorption due to surface plasmon resonance (SPR), tunable in NIR region	Au nanospheres, nanorods, nanoshells for targeted PA imaging and therapy^43,106^
iron oxide nanoparticles	magnetic properties, can be functionalized for multimodal imaging (MRI, PA)	used in combination with photothermal agents for targeted imaging and therapy^106^
silica nanoparticles	biocompatible, can encapsulate photosensitizers and drugs for theranostics	photosensitizer-loaded silica nanoshells for image-guided therapy and diagnosis^93,106^
carbon-based nanoparticles	high photothermal conversion, strong NIR absorption	carbon nanotubes and graphene-based nanoparticles for PA imaging and therapy^43,106^
chitosan nanoparticles	biodegradable, can be loaded with drugs or contrast agents	used as carriers for targeted delivery and imaging in CRC^106^
metal–organic frameworks (MOFs)	high porosity, tunable composition, can encapsulate drugs and imaging agents	MOF-based nanoparticles loaded with oxaliplatin and indocyanine green for PA imaging and therapy (example: Fe-MOFs)^107^
polymeric nanoparticles	biocompatible, can be engineered for controlled release and targeting	PEGylated and other polymer-coated nanoparticles for enhanced PA signal and tumor targeting^93,107^

To further clarify the integration of these technologies, Figure 6 represents a workflow summarizing how nanoparticle-enhanced imaging can be combined with machine learning analysis for improved colorectal cancer detection and characterization. In this approach, functionalized nanoparticles are first administered and selectively accumulate at tumor sites, enhancing imaging contrast for modalities such as photoacoustic imaging. The acquired images are then preprocessed and subjected to feature extraction, after which machine learning algorithms analyze these features to enable accurate tumor identification, classification, and treatment monitoring. This integrated workflow exemplifies the synergy between nanotechnology and artificial intelligence in advancing precision oncology.^53,102,103^ However, nanoparticle-enhanced PA imaging faces limitations such as potential toxicity, uncertain long-term biocompatibility, and challenges in large-scale synthesis and regulatory approval. Additionally, heterogeneous nanoparticle distribution and variable clearance rates in vivo can affect imaging accuracy and therapeutic outcomes.^104,105^

**Figure 6:**
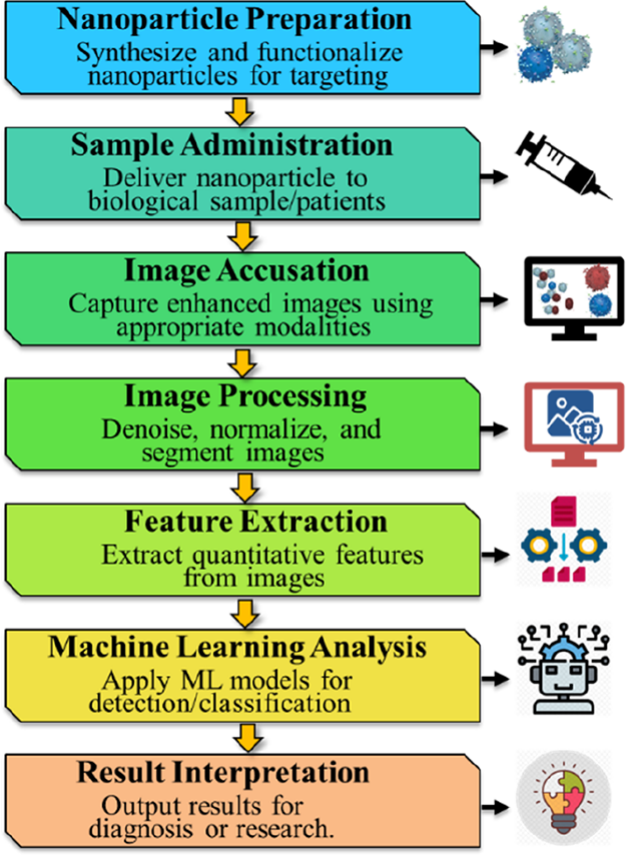
Flowchart showing nanoparticle-enhanced imaging with ML analysis.

### 2.5 Photoacoustic Machine Learning

Photoacoustic imaging combined with artificial intelligence offers a powerful approach to cancer diagnosis by integrating high-resolution functional imaging with advanced machine learning algorithms to enhance detection accuracy and classification.^43,108^ AI and machine learning have been effectively integrated with photoacoustic imaging to improve colorectal cancer detection by analyzing the spectral and textural differences between malignant and normal tissues. Using classifiers like Generalized Linear Models (GLM) and Support Vector Machines (SVM), features such as total hemoglobin concentration (rHbT) and specific spectral intensity patterns from coregistered PAT and US systems were extracted and evaluated. Image features like gray-level co-occurrence matrices (GLCMs) quantified texture differences, while spectral parameters like slope and intercept reflected the structural and vascular alterations in malignant tissues. Optimal AUC values for distinguishing cancerous from normal tissues were achieved by combining features like rHbT and spectral intensity. This ML-driven approach enhances diagnostic accuracy, offering potential applications in cancer screening, treatment monitoring, and surgical margin assessments, with plans to refine resolution and adapt the system for in vivo use.^44^ Similarly, ML can enhance photoacoustic imaging for colorectal cancer detection by improving signal quality and extracting essential molecular details. Adaptive Modified Kalman Filters (MKF) and backward Rauch-Tung-Striebel (BRTS) smoothers help reduce noise and correct signal distortions, leading to high-resolution imaging. PAI systems equipped with 532 nm lasers and 10 MHz ultrasound transducers can detect molecular markers like hemoglobin, revealing tumor-related hypoxia and vascular issues. ML-driven denoising improves the signal-to-noise ratio and image clarity, aiding in precisely visualizing colorectal tissue layers. Additionally, ML expands the capabilities of PAI by enabling more accurate boundary delineation and better tissue characterization. These developments provide a noninvasive, high-resolution approach for diagnosing and staging colorectal cancer, ultimately allowing for earlier detection and more targeted treatments.^109^ In addition to the above-described filters and smoothers, the employment of AI and Machine Learning-based techniques also contributes to the enhancement of PA imaging-based colorectal cancer detection via the development of different platforms, such as a coregistered ultrasound-photoacoustic (US-PAM) system. The integration of ML with PAM can significantly advance colorectal cancer detection by enhancing the precision of imaging and classification of the samples of interest. This system employs a DenseNet-based model to analyze coregistered US and PA images, capturing both structural and functional tissue information. The endorectal probe delivers high-resolution, 360° scans using a 1064 nm Nd:YAG laser and a single-element ring transducer with a 20 MHz center frequency. The DenseNet model processes five input channels, including coregistered US and PAM regions of interest (ROIs) and individualized references from normal tissue, which mitigate interpatient variability. The US-PAM DenseNet model demonstrated a significantly higher average accuracy of 89.1 ± 0.8% compared to the 75.1 ± 1.6% accuracy achieved by models trained on US images (Figure 7). Guided backpropagation further visualizes tumor regions, enhancing the system’s clinical interpretability. This dual-modality approach improves cancer detection, differentiates scars from residual tumors, and aids in real-time surgical decision-making (Figure 3).^65^ Similar to the previous study, Leng et al. developed a novel PAM/US endoscope, which was used for the imaging of rectal cancer after surgical interventions such as radiation and chemotherapy, as a way to manage post-treatment residual tumors. The PAM/US endoscopy system used deep-learning convolutional neural network (CNN) models, which analyzed photoacoustic and ultrasound data from both in vivo and ex vivo colorectal specimens. The study trained models on over 4600 regions of interest to differentiate normal and cancerous tissues with high accuracy, leveraging features such as vascular patterns and signal intensity. PAM-CNN models demonstrated superior diagnostic performance, achieving an AUC of 0.96, compared to PAM-GLM classifiers (AUC: 0.82). This approach enabled accurate pathological response prediction in rectal cancer, minimizing unnecessary surgeries and optimizing patient care. However, further validation with larger data sets and improved signal-to-noise ratios is necessary for broader clinical adoption.^77^ Moreover, the integration of quantum computing with machine learning is emerging as a transformative approach in medical decision-making, offering new possibilities for high-dimensional data processing and personalized diagnostics.^110^ PAI combined with ML has demonstrated significant advancements in colorectal cancer diagnosis by improving imaging precision, enhancing diagnostic accuracy, and enabling effective differentiation between normal and malignant tissues. These ML-driven systems, utilizing deep learning models and advanced denoising techniques, offer high-resolution, noninvasive diagnostic tools with potential applications in early detection, treatment monitoring, and surgical decision-making, providing a way of improvement for cancer research. Despite these advances, ML-enhanced photoacoustic imaging faces challenges such as the need for large, annotated data sets, potential overfitting, variability in imaging protocols, and the requirement for robust validation in diverse clinical settings before widespread adoption.^111,112^

**Figure 7:**
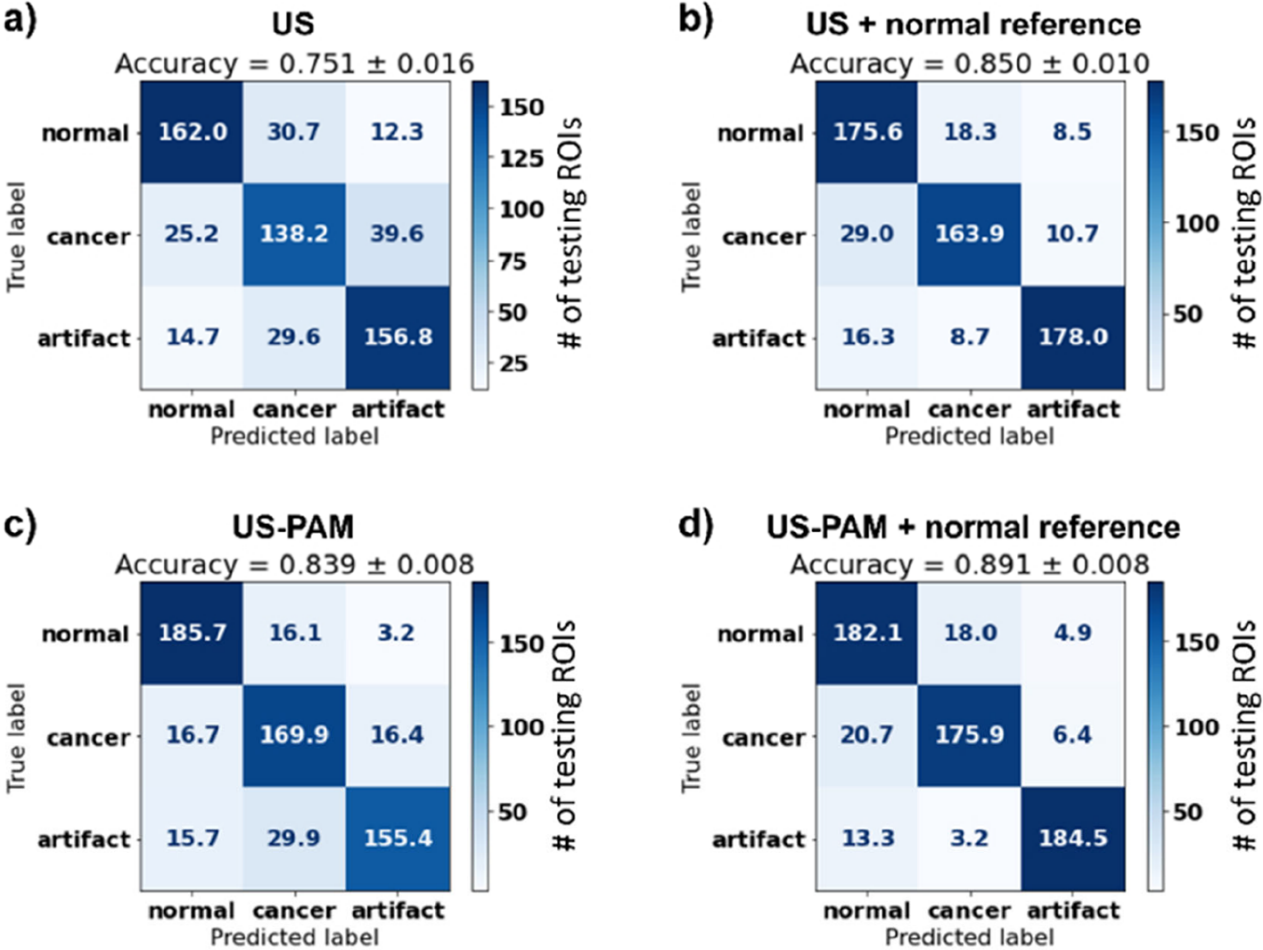
Three-class classification performance (normal, cancer, artifact) using confusion matrices for different model configurations. (a) US-only model achieved an accuracy of 75.1 ± 1.6%, showing significant misclassifications, particularly between cancer and artifact images. (b) Adding a normal reference to the US model improved accuracy to 85.0 ± 1.0%, reducing misclassifications between cancer and artifacts. (c) US-PAM model, which incorporates coregistered PA images, achieved 83.9 ± 0.8%, demonstrating better differentiation of normal images from cancer and artifacts. (d) Combining US-PAM with a normal reference achieved the highest accuracy of 89.1 ± 0.8%, with the most balanced performance and minimal misclassifications across all classes. These results emphasize the benefits of integrating coregistered PA images and a normal reference to improve classification accuracy. (Reproduced from ref 65. Available under an Optica Open Access Publishing Agreement. Copyright 2023, Optica Publishing Group.)

Photoacoustic integrated technologies are transforming the landscape of colorectal cancer detection and diagnosis. By harnessing the unique optical absorption properties of biomolecules such as hemoglobin, these innovations provide vital insights into tumor vascularization, oxygenation levels, and tissue morphology. Table 3 clearly illustrates the advancements in photoacoustic imaging techniques, showcasing various light sources, targeted biomolecules, and their impactful applications in CRC detection. These cutting-edge technologies not only enhance tumor detection but also precisely delineate tumor boundaries and evaluate therapeutic responses with remarkable sensitivity. With the integration of these powerful tools, clinicians can achieve earlier and more accurate diagnoses, significantly improving patient outcomes.

**Table 3:** Overview of Photoacoustic Integrated Imaging Technologies and Their Clinical Applications in Colorectal Cancer

photoacoustic integrated technology	sample used	light source	wavelength used (nm)	detector details	targeted biomolecules	clinical relevance in CRC	references
photoacoustic ultrasound	human colorectal tissue	Ti:sapphire laser optically pumped with Q-switched Nd-YAG laser	730, 780, 800, 830	endocavity ultrasound transducer array (6-MHz central frequency, 80% bandwidth)	oxy- and deoxy-hemoglobin	• enhanced tumor detection	^44^
						• achieves tissue penetration over 4–5 cm	
						• identifying tumor boundaries	
photoacoustic ultrasound	human colon tissue	acousticX LED system	850	A 7-MHz ultrasound probe		• it can differentiate between malignant, fatty, and healthy tissues	^62^
photoacoustic ultrasound	colorectal, esophageal, and gastric lesions	Alexandrite laser	750	linear transducer array with a central frequency of 9 MHz and 128 elements	hemoglobin	• enhances visualization of deep blood vessels	^63^
						• assists in evaluating chemotherapy response and drug delivery efficacy	
						• high Resolution and sensitivity of micro-vessels (10–50 μm)	
photoacoustic microscopy	human colorectal tissue	Nd:YAG pumped Ti:Sapphire laser	750	focused ring transducer (4.0 mm O.D., *f* = 12.7 mm, 20 MHz)	hemoglobin (vascular signal)	• provides cross-sectional imaging with morphological and vascular insights	^76^
						• identifies small and early lesions	
						• resolves tissue layers up to 8 mm depth	
photoacoustic microscopy	human colorectal tissue in vivo and ex vivo	diode-pumped, Q-switched Nd:YAG laser	1024	25 MHz central frequency and 15 mm focus transducer	hemoglobin (microvessel density)	• provides vascular details	^52^
						• non-invasive monitoring of vascular regrowth and tumor morphology after therapy	
photoacoustic nanotechnology	flank of Balb/c nude mice	NIR laser	808			• enables imaging-guided therapy for precise treatment monitoring	^95^
						• enhances tumor targeting via nanoparticle delivery with reduced systemic toxicity	
photoacoustic nanotechnology	armpit of nude mouse	NIR laser	808		endogenous hydrogen sulfide (H2S)	• PA imaging allowed precise visualization of tumors by leveraging strong NIR absorption of copper sulfide generated in situ	^100^
photoacoustic colonoscopy/endoscopy	pig colorectal tissue and human colorectal tissue	Nd-YAG laser	1065	ring transducer array with 6 MHz with a–6 dB bandwidth of 60%.	-	• enables non-invasive, high-contrast imaging of colorectal tissues.	^84^
						• provides 360° view imaging without rotating the transducer, leading to faster data acquisition	
	New Zealand white rabbit rectum (in vivo).	pulsed Q-switched laser	420 to 750 with an average 5 nm increment.	15 MHz polyvinylidene fluoride (PVDF) transducer with 150% bandwidth	oxy- and deoxy-hemoglobin	• provides both surface optical imaging and deep-tissue tomography	^85^
						• enables vascular structure imaging for microvascular abnormalities	
photoacoustic colonoscopy/endoscopy	New Zealand rabbits rectal (in-vivo and ex-vivo)	optical parametric oscillator (OPO) laser	760, 840	10 MHz planar ultrasonic transducer	indocyanine green, hemoglobin (Hb and HbO2)	• enables centimeter-scale imaging depth for detecting deep-seated tumors	^86^
						• provides sub-millimeter spatial resolution	
photoacoustic and machine learning	ex vivo colorectal tissue	pulsed laser	532	ultrasound transducer with a center frequency of 10 MHz.		• improve the quality of PA images	^109^
						• the AI algorithm effectively reduces noise and enhances signal clarity	
photoacoustic and machine learning:	human rectal tissue	Nd-YAG laser	1064	single-element ring transducer with a 20 MHz center frequency, 75% bandwidth, and 12.7 mm focal length		• improve the accuracy of identifying complete responders in rectal cancer	^65^
						• it enhances the differentiation of malignant from non-cancerous tissue	
photoacoustic and machine learning:	human rectal tissue in vivo and ex vivo	Nd-YAG laser	1064	20 MHz ultrasound transducer with 75% bandwidth		• enhances diagnostic accuracy in determining pathological complete response (pCR)	^77^
						• it can potentially reduce unnecessary surgery for those who have achieved pCR	

## 3 Machine Learning, Data Interpretation, and Their Current Challenges and Limitations

ML has become increasingly pivotal in colorectal CRC diagnostics, offering powerful tools for risk prediction, early detection, and clinical decision support. ML models, including artificial neural networks, support vector machines, random forests, and ensemble methods, have been widely applied to analyze electronic medical records, imaging data, and histopathological images, enabling the extraction of clinically relevant features that support early and accurate diagnosis.^113–115^ For example, recent studies have demonstrated that ML can achieve high sensitivity and specificity in predicting CRC and advanced colorectal polyps, with area under the receiver operating characteristic (AUROC) values ranging up to 0.883, sensitivity of 0.832, and specificity of 0.802 in validated models.^113^ ML-driven systems can assist clinicians by prioritizing high-risk patients for colonoscopy, reducing unnecessary procedures, and streamlining the selection of appropriate diagnostic tests.^114^ In imaging, ML algorithms, particularly convolutional neural networks (CNNs), have shown remarkable accuracy in analyzing endoscopic, histopathological, and multimodal imaging data, further enhancing diagnostic precision and enabling real-time image interpretation.^115,116^ These approaches not only improve diagnostic accuracy but also help minimize waiting times and healthcare costs by guiding efficient clinical workflows.^114^

Despite the significant promise of ML in CRC diagnosis, several challenges and limitations remain. The field is characterized by considerable heterogeneity in study methodologies, data sources, and model architectures, leading to variability in reported outcomes and making it difficult to standardize approaches across different clinical settings.^113,114^ Many ML models rely on retrospective data sets and numerical features, which may limit their effectiveness in early stage CRC prediction and their generalizability to diverse patient populations.^114,115^ Additionally, the integration of ML into routine clinical practice faces barriers related to data privacy, regulatory approval, and the need for robust validation in prospective studies.^113,114^ The quality and representativeness of training data are critical, as biases in data collection can result in models that underperform in real-world scenarios. Furthermore, while ML can enhance image interpretation and risk stratification, it cannot entirely replace clinical judgment or invasive diagnostic procedures such as colonoscopy, which remain the gold standard for CRC diagnosis.^113,114^ Ongoing research is needed to address these limitations, improve model interpretability, and ensure that ML-driven tools are both clinically effective and accessible to healthcare providers.

## 4 Regulatory Considerations

The integration of PA imaging with other modalities and advancing diagnostic technologies into clinical practice for colorectal cancer requires careful regulatory oversight to ensure patient safety, efficacy, and data protection. Regulatory agencies, including the U.S. Food and Drug Administration (FDA), European Medicines Agency (EMA), and national health authorities, mandate rigorous validation and approval processes for diagnostic devices and software algorithms before their adoption in routine care. These processes typically involve multicentric clinical trials to assess diagnostic accuracy, reproducibility, and potential risks associated with technology.^117,118^ Additionally, the use of artificial intelligence and machine learning in medical imaging introduces challenges related to data privacy, algorithm transparency, and ongoing monitoring for bias or performance drift, all of which must be addressed to meet regulatory standards and ensure equitable patient outcomes.^117^ Guidelines from leading organizations, such as the European Society of Gastrointestinal and Abdominal Radiology (ESGAR) and the American College of Radiology (ACR), emphasize the importance of evidence-based practice and standardized reporting to support regulatory compliance and facilitate the safe implementation of new technologies in colorectal cancer diagnosis and management.^117,119^

## 5 Challenges and Potential Solutions

PAI encounters significant obstacles that hinder its optimal application in CRC diagnostics. One major issue is the restricted imaging depth, primarily due to light scattering within biological tissues, which diminishes its ability to visualize deeper-seated lesions.^120^ However, innovative solutions, such as employing multiwavelength lasers that leverage specific optical windows to quantify chromophore concentrations, have shown promise in enhancing tissue penetration.^121^

Another challenge is motion artifacts, which result from patient or organ movement during imaging and can significantly degrade image quality and spatial accuracy.^122^ These artifacts are particularly problematic in abdominal imaging due to respiratory and peristaltic motion. To address this, advanced stabilization strategies have been proposed, including mechanical gating, breath-hold techniques, and real-time motion correction algorithms.^123^ Recently, AI-driven predictive models have shown promise in anticipating motion patterns and dynamically compensating for them during acquisition.^124^ However, these methods are still under development and face limitations related to processing speed, generalizability across patients, and integration with existing clinical systems.

The high cost and intricate design of PAI systems also limit their widespread availability. Researchers are focusing on creating portable, affordable solutions, incorporating streamlined hardware designs and compact laser technology to make PAI accessible, even in resource-constrained environments.^125,126^ A significant concern regarding the use of nanoparticle-based contrast agents in PAI is their potential toxicity and the possibility of triggering immune responses, which restricts their widespread clinical implementation. To mitigate these risks, thorough safety evaluations, the approval of biocompatible materials, and in-depth toxicity studies are crucial steps to ensure their safe use in patients.^94^

Additionally, the absence of standardized imaging protocols leads to inconsistencies in PAI results, hindering the reproducibility and scalability of technology. Establishing global, standardized imaging procedures and protocols through global collaborative efforts is necessary to ensure reliable and comparable data across different research settings and clinical applications.^127^ The integration of PAI with other imaging modalities also introduces challenges related to harmonizing data from different sources, mainly due to discrepancies in spatial resolution and imaging speed.^128^ Utilizing sophisticated computational techniques, especially AI-based algorithms, can facilitate the fusion of multimodal data, providing a more comprehensive understanding of colorectal cancer.^108,129^ Moreover, the inherent complexity of interpreting the high-dimensional data sets produced by PAI calls for the development of automated tools. By leveraging machine learning models trained on combined data sets from various imaging techniques, meaningful insights can be extracted more efficiently, enabling more accurate diagnostics and treatment decisions. Tackling these obstacles collectively will enhance the clinical utility of PAS in the diagnosis, personalized treatment, and management of colorectal cancer.^54,130^ All the challenges in PA imaging for CRC diagnosis and their potential solutions are summarized in Table 4.

**Table 4:** Challenges in PA Imaging for CRC Diagnosis and Their Potential Solutions

challenge	description	potential solution	reference(s)
limited imaging Depth	scattering of light in tissue limits visualization of deep lesions.	use of multiwavelength lasers within optimal optical windows to improve penetration.	^120,121^
motion artifacts	movement during imaging (e.g., breathing, peristalsis) degrades image quality.	mechanical gating, breath-holding, real-time motion correction, and AI-based motion prediction.	^122–124^
high cost and complex system design	limits deployment in clinical and resource-limited settings.	development of portable and affordable PAI systems using compact lasers and simplified hardware.	^125,126^
nanoparticle toxicity	risk of immune response and toxicity restricts clinical use.	biocompatibility testing, safer contrast agents, and rigorous toxicity assessments.	^94^
lack of standardized protocols	inconsistent imaging results and reproducibility issues.	development of global, standardized imaging protocols through collaborative efforts.	^127^
integration with other modalities	difficulties in harmonizing data with varying resolution and speed.	use of AI-based algorithms for multimodal data fusion.	^108,128,129^
high-dimensional data interpretation	complexity in analyzing rich data outputs from PAI systems.	machine learning-based automated tools for efficient data processing and clinical insight generation.	^54,130^

## 6 Future Outlook

Future innovations in PAI aim to address current challenges such as limited imaging depth, motion artifacts, and the high cost and complexity of existing systems. Advancements in system miniaturization and the development of portable, cost-effective devices will support the deployment of PAI in point-of-care and low-resource settings. Real-time integration with other imaging modalities such as CT, MRI, and PET may further enhance diagnostic accuracy by offering comprehensive anatomical and molecular information. Progress in nanoparticle engineering will improve molecular specificity, biosafety, and therapeutic delivery, expanding the potential for image-guided therapies. In parallel, global collaborative initiatives, such as shared databases and federated learning frameworks, will accelerate the development of robust, generalizable AI models for automated analysis. Integration with liquid biopsy techniques and continuous improvements in PAI-enabled endoscopy will expand the scope of noninvasive diagnostics and treatment monitoring. As technological, regulatory, and ethical challenges are progressively overcome, PAI is poised to transform CRC care by enabling early detection, precise monitoring, and personalized therapeutic strategies, ultimately improving patient outcomes and redefining standards in cancer diagnostics and management.

## 7 Conclusions

The integration of PAI with multimodal strategies represents a pivotal advancement in the detection, diagnosis, and management of CRC. Leveraging techniques such as photoacoustic PAT, PAM and endoscopic photoacoustic imaging, PAI provides high-resolution insights into tissue morphology, vascular architecture, and functional parameters like oxygen saturation. When combined with complementary imaging modalities such as ultrasound and enhanced by nanoparticle-based targeting, PAI enables contrast-rich, anatomically and functionally informative imaging of both superficial and deep-seated tissues. Each modality contributes unique advantages, PAT for volumetric imaging, PAM for localized high-resolution imaging, and endoscopic PAI for minimally invasive, real-time diagnostics. The integration of AI further refines these capabilities by facilitating data interpretation, reducing noise, and extracting clinically relevant biomarkers. Collectively, these advancements lay the foundation for precision diagnostics and personalized treatment planning in colorectal cancer.

## References

[1] Ferlay, J. ; Ervik, M. ; Lam, F. ; Laversanne, M. ; Colombet, M. ; Mery, L. ; Piñeros, M. ; Znaor, A. ; Soerjomataram, I. ; Bray, F. Cancer Today. https://gco.iarc.who.int/today/ (accessed 2025-05-07).

[2] Sathishkumar K., Chaturvedi M., Das P., Stephen S., Mathur P. (2022). Cancer Incidence
Estimates for 2022 & Projection for 2025: Result from National
Cancer Registry Programme, India. Indian J.
Med. Res..

[3] Khazaee
Fadafen M., Rezaee K. (2023). Ensemble-Based Multi-Tissue Classification
Approach of Colorectal Cancer Histology Images Using a Novel Hybrid
Deep Learning Framework. Sci. Rep..

[4] Siegel R. L., Kratzer T. B., Giaquinto A. N., Sung H., Jemal A. (2025). Cancer Statistics,
2025. Ca.

[5] Roshandel G., Ghasemi-Kebria F., Malekzadeh R. (2024). Colorectal Cancer: Epidemiology,
Risk Factors, and Prevention. Cancers.

[6] Dekker E., Tanis P. J., Vleugels J. L. A., Kasi P. M., Wallace M. B. (2019). Colorectal
Cancer. Lancet.

[7] Centelles J. J. (2012). General
Aspects of Colorectal Cancer. Int. Sch. Res.
Not..

[8] Nguyen L. H., Goel A., Chung D. C. (2020). Pathways
of Colorectal Carcinogenesis. Gastroenterology.

[9] Yamane L., Scapulatempo-Neto C., Reis R. M., Guimarães D. P. (2014). Serrated
Pathway in Colorectal Carcinogenesis. World
J. Gastroenterol. WJG.

[10] Yang C., Xiang E., Chen P., Fang X. (2024). Evolutionary History
of Adenomas to Colorectal Cancer in FAP Families. Front. Genet..

[11] Leggett B., Whitehall V. (2010). Role of the Serrated Pathway in Colorectal
Cancer Pathogenesis. Gastroenterology.

[12] Moufarrij S., Gazzo A., Rana S., Selenica P., Abu-Rustum N. R., Ellenson L. H., Liu Y. L., Weigelt B., Momeni-Boroujeni A. (2024). Concurrent *POLE* Hotspot Mutations and Mismatch Repair Deficiency/Microsatellite
Instability in Endometrial Cancer: A Challenge in Molecular Classification. Gynecol. Oncol..

[13] Aiderus A., Barker N., Tergaonkar V. (2024). Serrated Colorectal
Cancer: Preclinical
Models and Molecular Pathways. Trends Cancer.

[14] Pierantoni C., Cosentino L., Ricciardiello L. (2024). Molecular Pathways of Colorectal
Cancer Development: Mechanisms of Action and Evolution of Main Systemic
Therapy Compunds. Dig. Dis..

[15] Li C., Du X., Zhang H., Liu S. (2024). Knockdown of Ribosomal Protein L22-like
1 Arrests the Cell Cycle and Promotes Apoptosis in Colorectal Cancer. Cytojournal.

[16] Kanth P., Inadomi J. M. (2021). Screening and Prevention
of Colorectal Cancer. BMJ..

[17] Dennis R., Tou S., Miller R. (2011). Colorectal
Cancer: Prevention and Early Diagnosis. Medicine
(Baltimore).

[18] Wang K., Ning S., Zhang S., Jiang M., Huang Y., Pei H., Li M., Tan F. (2025). Extracellular
Matrix Stiffness Regulates
Colorectal Cancer Progression via HSF4. J. Exp.
Clin. Cancer Res..

[19] Tong G., Peng T., Chen Y., Sha L., Dai H., Xiang Y., Zou Z., He H., Wang S. (2022). Effects of
GLP-1 Receptor Agonists on Biological Behavior of Colorectal Cancer
Cells by Regulating PI3K/AKT/mTOR Signaling Pathway. Front. Pharmacol..

[20] Tonini V., Zanni M. (2024). Why Is Early Detection of Colon Cancer Still Not Possible in 2023?. World J. Gastroenterol..

[21] Vega P., Valentín F., Cubiella J. (2015). Colorectal Cancer Diagnosis: Pitfalls
and Opportunities. World J. Gastrointest. Oncol..

[22] Bhat S. K., East J. E. (2015). Colorectal Cancer: Prevention and Early Diagnosis. Medicine (Baltimore).

[23] Young P. E., Womeldorph C. M. (2013). Colonoscopy
for Colorectal Cancer Screening. J. Cancer.

[24] Stauffer, C. M. ; Pfeifer, C. Colonoscopy. In StatPearls; StatPearls Publishing: Treasure Island (FL), 2024.

[25] Mazouji O., Ouhajjou A., Incitti R., Mansour H. (2021). Updates on Clinical
Use of Liquid Biopsy in Colorectal Cancer Screening, Diagnosis, Follow-Up,
and Treatment Guidance. Front. Cell Dev. Biol..

[26] Wang K. S., Yu G., Xu C., Meng X. H., Zhou J., Zheng C., Deng Z., Shang L., Liu R., Su S., Zhou X., Li Q., Li J., Wang J., Ma K., Qi J., Hu Z., Tang P., Deng J., Qiu X., Li B. Y., Shen W. D., Quan R. P., Yang J. T., Huang L. Y., Xiao Y., Yang Z. C., Li Z., Wang S. C., Ren H., Liang C., Guo W., Li Y., Xiao H., Gu Y., Yun J. P., Huang D., Song Z., Fan X., Chen L., Yan X., Li Z., Huang Z. C., Huang J., Luttrell J., Zhang C. Y., Zhou W., Zhang K., Yi C., Wu C., Shen H., Wang Y. P., Xiao H. M., Deng H. W. (2021). Accurate
Diagnosis of Colorectal Cancer Based on Histopathology Images Using
Artificial Intelligence. BMC Med..

[27] Goede S. L., Rabeneck L., van Ballegooijen M., Zauber A. G., Paszat L. F., Hoch J. S., Yong J. H. E., Kroep S., Tinmouth J., Lansdorp-Vogelaar I. (2017). Harms, Benefits
and Costs of Fecal Immunochemical Testing
versus Guaiac Fecal Occult Blood Testing for Colorectal Cancer Screening. PLoS One.

[28] D’Souza N., Hicks G., Benton S., Abulafi M. (2020). The Diagnostic Accuracy
of the Faecal Immunochemical Test for Colorectal Cancer in Risk-Stratified
Symptomatic Patients. Ann. R. Coll. Surg. Engl..

[29] Beniwal S. S., Lamo P., Kaushik A., Lorenzo-Villegas D. L., Liu Y., MohanaSundaram A. (2023). Current Status and Emerging Trends
in Colorectal Cancer Screening and Diagnostics. Biosensors.

[30] Dhaliwal A., Vlachostergios P. J., Oikonomou K. G., Moshenyat Y. (2015). Fecal DNA
Testing for Colorectal Cancer Screening: Molecular Targets and Perspectives. World J. Gastrointest. Oncol..

[31] Kadari M., Subhan M., Saji Parel N., Krishna P. V., Gupta A., Uthayaseelan K., Uthayaseelan K., Sunkara N. A. B. S. (2022). CT Colonography
and Colorectal Carcinoma: Current Trends and Emerging Developments. Cureus.

[32] Van
Cutsem E., Verheul H. M. W., Flamen P., Rougier P., Beets-Tan R., Glynne-Jones R., Seufferlein T. (2016). Imaging in
Colorectal Cancer: Progress and Challenges for the Clinicians. Cancers.

[33] Riccioni M. E., Urgesi R., Cianci R., Bizzotto A., Spada C., Costamagna G. (2012). Colon Capsule Endoscopy: Advantages, Limitations and
Expectations. Which Novelties? World. J. Gastrointest.
Endosc..

[34] Hausmann J., Tal A., Gomer A., Philipper M., Moog G., Hohn H., Hesselbarth N., Plass H., Albert J., Finkelmeier F. (2021). Colon Capsule
Endoscopy: Indications, Findings, and Complications – Data
from a Prospective German Colon Capsule Registry Trial (DEKOR). Clin. Endosc..

[35] Ding J., Li Q., Lin J., He S., Chen W., He Q., Zhang Q., Chen J., Wu T., Zhong S., Li D. (2021). Optical Coherence Tomography for
the Early Detection of Colorectal
Dysplasia and Cancer: Validation in a Murine Model. Quant. Imaging Med. Surg..

[36] Schulte B., Göb M., Singh A. P., Lotz S., Draxinger W., Heimke M., Pieper M., Heinze T., Wedel T., Rahlves M., Huber R., Ellrichmann M. (2024). High-Resolution
Rectoscopy Using MHz Optical Coherence Tomography: A Step towards
Real Time 3D Endoscopy. Sci. Rep..

[37] Galema H. A., Meijer R. P. J., Lauwerends L. J., Verhoef C., Burggraaf J., Vahrmeijer A. L., Hutteman M., Keereweer S., Hilling D. E. (2022). Fluorescence-Guided
Surgery in Colorectal Cancer; A
Review on Clinical Results and Future Perspectives. Eur. J. Surg. Oncol. J. Eur. Soc. Surg. Oncol. Br. Assoc.
Surg. Oncol..

[38] Daniluk P., Mazur N., Swierblewski M., Chand M., Diana M., Polom K. (2022). Fluorescence Imaging
in Colorectal Surgery: An Updated Review and
Future Trends. Surg. Innov..

[39] Fadel M. G., Zonoobi E., Rodríguez-Luna M. R., Mishima K., Ris F., Diana M., Vahrmeijer A. L., Perretta S., Ashrafian H., Fehervari M. (2024). Efficacy and
Safety of Fluorescence-Guided Surgery
Compared to Conventional Surgery in the Management of Colorectal Cancer:
A Systematic Review and Meta-Analysis. Cancers.

[40] Liu Y., Nie L., Chen X. (2016). Photoacoustic
Molecular Imaging: From Multiscale Biomedical
Applications towards Early-Stage Theranostics. Trends Biotechnol..

[41] Ko C. W., Doria-Rose V. P., Barrett M. J., Kamineni A., Enewold L., Weiss N. S. (2019). Screening Flexible Sigmoidoscopy
versus Colonoscopy
for Reduction of Colorectal Cancer Mortality. Int. J. Colorectal Dis..

[42] Niedermaier T., Weigl K., Hoffmeister M., Brenner H. (2018). Flexible Sigmoidoscopy
in Colorectal Cancer Screening: Implications of Different Colonoscopy
Referral Strategies. Eur. J. Epidemiol..

[43] Mallidi S., Luke G. P., Emelianov S. (2011). Photoacoustic Imaging in Cancer Detection,
Diagnosis, and Treatment Guidance. Trends Biotechnol..

[44] Yang G., Amidi E., Chapman W. C., Nandy S., Mostafa A., Abdelal H., Alipour Z., Chatterjee D., Mutch M., Zhu Q. (2019). Co-Registered Photoacoustic and Ultrasound
Imaging of Human Colorectal Cancer. J. Biomed.
Opt..

[45] Huang, B. ; An, H. ; Gui, M. ; Qiu, Y. ; Xu, W. ; Chen, L. ; Li, Q. ; Yao, S. ; Lin, S. ; Khrustaleva, T. A. ; Wang, R. ; Lin, J. Qingjie Fuzheng Granule Prevents Colitis-Associated Colorectal Cancer by Inhibiting Abnormal Activation of NOD2/NF-κB Signaling Pathway Mediated by Gut Microbiota Disorder. Chin. Herb. Med. 2025. 10.1016/j.chmed.2025.04.001. PMC12301919 40734913

[46] Han X., Zhao C., Wang S., Pan Z., Jiang Z., Tang X. (2022). Multifunctional TiO2/C Nanosheets
Derived from 3D Metal-Organic Frameworks
for Mild-Temperature-Photothermal-Sonodynamic-Chemodynamic Therapy
under Photoacoustic Image Guidance. J. Colloid
Interface Sci..

[47] Wang Y., Xu Y., Song J., Liu X., Liu S., Yang N., Wang L., Liu Y., Zhao Y., Zhou W., Zhang Y. (2024). Tumor Cell-Targeting and Tumor Microenvironment-Responsive
Nanoplatforms
for the Multimodal Imaging-Guided Photodynamic/Photothermal/Chemodynamic
Treatment of Cervical Cancer. Int. J. Nanomed..

[48] He B., Wang L., Zhou W., Liu H., Wang Y., Lv K., He K. (2025). A Fusion Model to Predict the Survival of Colorectal
Cancer Based on Histopathological Image and Gene Mutation. Sci. Rep..

[49] Patel P., Hardik M., Patel P. (2013). A Review on Photoacoustic
Spectroscopy. Int. J. Pharm. Erud..

[50] Priya, M. Pulsed Laser Induced Photoacoustic Spectroscopy PLPAS of the Changes in Breast Tumor Microenvironment and Tumor Growth an Ex Vivo Experimental Study; Manipal University, 2016.

[51] Chohan D. P., Biswas S., Wankhede M., P Menon A. K., Basha S., Rodrigues J., Mukunda D. C., Mahato K. K. (2024). Assessing
Breast
Cancer through Tumor Microenvironment Mapping of Collagen and Other
Biomolecule Spectral Fingerprints—A Review. ACS Sens..

[52] Kou S., Thakur S., Eltahir A., Nie H., Zhang Y., Song A., Hunt S. R., Mutch M. G., Chapman W. C., Zhu Q. (2024). A Portable Photoacoustic Microscopy
and Ultrasound System for Rectal
Cancer Imaging. Photoacoustics.

[53] Yan J., Wang C., Jiang X., Wei Y., Wang Q., Cui K., Xu X., Wang F., Zhang L. (2021). Application of Phototherapeutic-Based
Nanoparticles in Colorectal Cancer. Int. J.
Biol. Sci..

[54] Yin Z., Yao C., Zhang L., Qi S. (2023). Application of Artificial Intelligence
in Diagnosis and Treatment of Colorectal Cancer: A Novel Prospect. Front. Med..

[55] Xia J., Yao J., Wang L. V. (2014). Photoacoustic Tomography: Principles
and Advances. Electromagn. Waves Camb. Mass.

[56] Park J., Choi S., Knieling F., Clingman B., Bohndiek S., Wang L. V., Kim C. (2024). Clinical Translation
of Photoacoustic
Imaging. Nat. Rev. Bioeng..

[57] Yu Y., Feng T., Qiu H., Gu Y., Chen Q., Zuo C., Ma H. (2024). Simultaneous Photoacoustic and Ultrasound Imaging:
A Review. Ultrasonics.

[58] Grasso V., Hassan H. W., Mirtaheri P., Willumeit-Römer R., Jose J. (2022). Recent Advances in
Photoacoustic Blind Source Spectral Unmixing Approaches
and the Enhanced Detection of Endogenous Tissue Chromophores. Front. Signal Process..

[59] Zhang Y., Jeon M., Rich L. J., Hong H., Geng J., Zhang Y., Shi S., Barnhart T. E., Alexandridis P., Huizinga J. D., Seshadri M., Cai W., Kim C., Lovell J. F. (2014). Non-Invasive Multimodal Functional
Imaging of the Intestine
with Frozen Micellar Naphthalocyanines. Nat.
Nanotechnol..

[60] Wang L. V., Hu S. (2012). Photoacoustic Tomography. In Vivo Imaging from Organelles to Organs. Science.

[61] Wang L. V., Yao J. (2016). A Practical Guide to
Photoacoustic Tomography in the Life Sciences. Nat. Methods.

[62] Ghanbarzadeh-Daghyean, A. ; Joseph, F. K. ; Mooij, C. ; Van Der Stel, S. ; Ruers, T. Application of a Photoacoustic Sensor for Colon Cancer Imaging: A Case Report. In 2023 IEEE Sensors; IEEE: Vienna, Austria, 2023; pp. 1–3.

[63] Ikematsu H., Ishihara M., Okawa S., Minamide T., Mitsui T., Kuwata T., Ito M., Kinoshita T., Fujita T., Yano T., Omori T., Ozawa S., Murakoshi D., Irisawa K., Ochiai A. (2022). Photoacoustic Imaging
of Fresh Human Surgically and Endoscopically Resected Gastrointestinal
Specimens. DEN Open.

[64] Xu J., Lv Z., Wang L., Wu X., Tan B., Shen X.-C., Chen H. (2024). Tuning Tumor Targeting
and Ratiometric Photoacoustic Imaging by Fine-Tuning
Torsion Angle for Colorectal Liver Metastasis Diagnosis. Chem. −Eur. J..

[65] Lin Y., Kou S., Nie H., Luo H., Eltahir A., Chapman W., Hunt S., Mutch M., Zhu Q. (2023). Deep Learning Based
on Co-Registered Ultrasound and Photoacoustic Imaging Improves the
Assessment of Rectal Cancer Treatment Response. Biomed. Opt. Express.

[66] Zhang J., Duan F., Liu Y., Nie L. (2020). High-Resolution Photoacoustic
Tomography for Early-Stage Cancer Detection and Its Clinical Translation. Radiol. Imaging Cancer.

[67] Lee H., Choi W., Kim C., Park B., Kim J. (2023). Review on
Ultrasound-Guided Photoacoustic Imaging for Complementary Analyses
of Biological Systems in Vivo. Exp. Biol. Med..

[68] Nyayapathi N., Zheng E., Zhou Q., Doyley M., Xia J. (2024). Dual-Modal
Photoacoustic and Ultrasound Imaging: From Preclinical to Clinical
Applications. Front. Photonics.

[69] Choi S., Kim J., Jeon H., Kim C., Park E.-Y. (2025). Advancements in
Photoacoustic Detection Techniques for Biomedical Imaging. Npj Acoust..

[70] Yao J., Wang L. V. (2013). Photoacoustic Microscopy. Laser
Photonics Rev..

[71] Li W., Lv J., Li H., Song L., Zhang R., Zhao X., Xuan F., Sun T., Long K., Zhao Y., Nie L. (2025). Quantification of Vascular Remodeling and Sinusoidal Capillarization
to Assess Liver Fibrosis with Photoacoustic Imaging. Radiology.

[72] Liu W., Yao J. (2018). Photoacoustic Microscopy: Principles and Biomedical Applications. Biomed. Eng. Lett..

[73] Zhu X., Menozzi L., Cho S.-W., Yao J. (2024). High Speed Innovations
in Photoacoustic Microscopy. Npj Imaging.

[74] Hysi E., Moore M. J., Strohm E. M., Kolios M. C. (2021). A Tutorial in Photoacoustic
Microscopy and Tomography Signal Processing Methods. J. Appl. Phys..

[75] Zhang J., Shi Y., Zhang Y., Liu H., Li S., Liu L. (2024). Resolution
Enhancement Strategies in Photoacoustic Microscopy: A Comprehensive
Review. Micromachines.

[76] Leng X., Chapman W., Rao B., Nandy S., Chen R., Rais R., Gonzalez I., Zhou Q., Chatterjee D., Mutch M., Zhu Q. (2018). Feasibility
of Co-Registered Ultrasound
and Acoustic-Resolution Photoacoustic Imaging of Human Colorectal
Cancer. Biomed. Opt. Express.

[77] Leng X., Amidi E., Kou S., Cheema H., Otegbeye E., Chapman W. J., Mutch M., Zhu Q. (2021). Rectal Cancer Treatment
Management: Deep-Learning Neural Network Based on Photoacoustic Microscopy
Image Outperforms Histogram-Feature-Based Classification. Front. Oncol..

[78] Yao J., Kim C., Kolios M., Hu S. (2023). Editorial: Breaking
the Speed Limits
in Photoacoustic Microscopy. Photoacoustics.

[79] Loc I., Unlu M. B. (2024). Accelerating Photoacoustic
Microscopy by Reconstructing
Undersampled Images Using Diffusion Models. Sci. Rep..

[80] Hazewinkel Y., Dekker E. (2011). Colonoscopy: Basic Principles and Novel Techniques. Nat. Rev. Gastroenterol. Hepatol..

[81] Yoon T.-J., Cho Y.-S. (2013). Recent Advances
in Photoacoustic Endoscopy. World J. Gastrointest.
Endosc..

[82] He H., Englert L., Ntziachristos V. (2023). Optoacoustic Endoscopy of the Gastrointestinal
Tract. ACS Photonics.

[83] Kim J., Heo D., Cho S., Ha M., Park J., Ahn J., Kim M., Kim D., Jung D. H., Kim H. H., Kim H. M., Kim C. (2024). Enhanced Dual-Mode
Imaging: Superior Photoacoustic and Ultrasound
Endoscopy in Live Pigs Using a Transparent Ultrasound Transducer. Sci. Adv..

[84] Yuan Y., Yang S., Xing D. (2010). Preclinical
Photoacoustic Imaging
Endoscope Based on Acousto-Optic Coaxial System Using Ring Transducer
Array. Opt. Lett..

[85] Liu N., Yang S., Xing D. (2018). Photoacoustic
and Hyperspectral Dual-Modality
Endoscope. Opt. Lett..

[86] Jiang J., Yuan C., Zhang J., Xie Z., Xiao J. (2023). Spectroscopic
Photoacoustic/Ultrasound/Optical-Microscopic Multimodal Intrarectal
Endoscopy for Detection of Centimeter-Scale Deep Lesions. Front. Bioeng. Biotechnol..

[87] Wei N., Chen H., Li B., Dong X., Wang B. (2024). Advances in
Photoacoustic Endoscopic Imaging Technology for Prostate Cancer Detection. Photonics.

[88] Liang Y., Fu W., Li Q., Chen X., Sun H., Wang L., Jin L., Huang W., Guan B.-O. (2022). Optical-Resolution
Functional Gastrointestinal
Photoacoustic Endoscopy Based on Optical Heterodyne Detection of Ultrasound. Nat. Commun..

[89] Siddique S., Chow J. C. L. (2020). Application of Nanomaterials in Biomedical
Imaging
and Cancer Therapy. Nanomaterials.

[90] Siddique S., Chow J. C. L. (2022). Recent Advances
in Functionalized Nanoparticles in
Cancer Theranostics. Nanomaterials.

[91] Chow J. C. L. (2025). Nanomaterial-Based
Molecular Imaging in Cancer: Advances in Simulation and AI Integration. Biomolecules.

[92] Gogoi P., Kaur G., Singh N. K. (2022). Nanotechnology for Colorectal Cancer
Detection and Treatment. World J. Gastroenterol..

[93] Brar B., Ranjan K., Palria A., Kumar R., Ghosh M., Sihag S., Minakshi P. (2021). Nanotechnology in Colorectal Cancer
for Precision Diagnosis and Therapy. Front.
Nanotechnol..

[94] Neelamraju P. M., Gundepudi K., Sanki P. K., Busi K. B., Mistri T. K., Sangaraju S., Dalapati G. K., Ghosh K. K., Ghosh S., Ball W. B., Chakrabortty S. (2024). Potential Applications for Photoacoustic
Imaging Using Functional Nanoparticles: A Comprehensive Overview. Heliyon.

[95] Liu H., Xu C., Meng M., Li S., Sheng S., Zhang S., Ni W., Tian H., Wang Q. (2022). Metal-Organic
Framework-Mediated
Multifunctional Nanoparticles for Combined Chemo-Photothermal Therapy
and Enhanced Immunotherapy against Colorectal Cancer. Acta Biomater..

[96] Zhang S., Cai S., Ma Y. (2018). Association between *Fusobacterium Nucleatum* and Colorectal Cancer: Progress
and Future Directions. J. Cancer.

[97] Xu L., Hu B., He J., Fu X., Liu N. (2025). Intratumor Microbiome-Derived
Butyrate Promotes Chemo-Resistance in Colorectal Cancer. Front. Pharmacol..

[98] Bhole R. (2021). A Comprehensive
Review on Photodynamic Therapy (PDT) and Photothermal Therapy (PTT)
for Cancer Treatment. Turk. J. Oncol..

[99] Lv Z., He S., Wang Y., Zhu X. (2021). Noble Metal Nanomaterials for NIR-Triggered
Photothermal Therapy in Cancer. Adv. Healthc.
Mater..

[100] Xin Y., Yu Y., Su M., Li X., Elsabahy M., Gao H. (2023). In Situ-Activated Photothermal Nanoplatform for on-Demand NO Gas
Delivery and Enhanced Colorectal Cancer Treatment. J. Controlled Release.

[101] Li Q., Chen K., Huang W., Ma H., Zhao X., Zhang J., Zhang Y., Fang C., Nie L. (2021). Minimally
Invasive Photothermal Ablation Assisted by Laparoscopy as an Effective
Preoperative Neoadjuvant Treatment for Orthotopic Hepatocellular Carcinoma. Cancer Lett..

[102] Hester S. C., Kuriakose M., Nguyen C. D., Mallidi S. (2020). Role of Ultrasound
and Photoacoustic Imaging in Photodynamic Therapy for Cancer. Photochem. Photobiol..

[103] Lee H., Han S., Kye H., Kim T.-K., Choi W., Kim J. (2023). A Review on the Roles
of Photoacoustic Imaging for Conventional and
Novel Clinical Diagnostic Applications. Photonics.

[104] Han S., Ninjbadgar T., Kang M., Kim C., Kim J. (2023). Recent Advances
in Photoacoustic Agents for Theranostic Applications. Nanomaterials.

[105] Li X., Pan Z., Xiang C., Yuan Y., Chen J., Qing G., Ma J., Liang X.-J., Wu Y., Guo W. (2021). Structure Transformable Nanoparticles for Photoacoustic Imaging-Guided
Photothermal Ablation of Tumors *via* Enzyme-Induced
Multistage Delivery. Chem. Eng. J..

[106] Ghorbani F., Kokhaei P., Ghorbani M., Eslami M. (2020). Application
of Different Nanoparticles in the Diagnosis of Colorectal Cancer. Gene Rep..

[107] Farzam O. R., Mehran N., Bilan F., Aghajani E., Dabbaghipour R., Shahgoli G. A., Baradaran B. (2023). Nanoparticles
for Imaging-Guided Photothermal Therapy of Colorectal Cancer. Heliyon.

[108] Xu M., Chen Z., Zheng J., Zhao Q., Yuan Z. (2023). Artificial
Intelligence-Aided Optical Imaging for Cancer Theranostics. Semin. Cancer Biol..

[109] Hu T., Huang Z., Ge P., Gao F., Gao F. (2023). Adaptive Denoising
of Photoacoustic Signal and Image Based on Modified Kalman Filter. J. Biophotonics.

[110] Chow J. C. L. (2025). Quantum Computing and Machine Learning
in Medical Decision-Making:
A Comprehensive Review. Algorithms.

[111] Wang R., Zhu J., Xia J., Yao J., Shi J., Li C. (2023). Photoacoustic Imaging with Limited
Sampling: A Review
of Machine Learning Approaches. Biomed. Opt.
Express.

[112] Yang C., Lan H., Gao F., Gao F. (2021). Review of
Deep Learning for Photoacoustic Imaging. Photoacoustics.

[113] Malik, S. ; Naqvi, A. ; Tenorio, B. G. ; Farrukh, F. ; Tariq, R. ; Adler, D. G. Machine Learning for Predicting Colorectal Cancer and Advanced Colorectal Polyps: A Systematic Review and Meta-Analysis. J. Clin. Gastroenterol. 2025. 10.1097/MCG.0000000000002172. 40434809

[114] Minh N. H., Quy T. Q., Tam N. D., Tuan T. M., Son L. H. (2025). A Practical Approach for Colorectal
Cancer Diagnosis
Based on Machine Learning. PLoS One.

[115] Ning B., Chi J., Meng Q., Jia B. (2024). Accurate Prediction
of Colorectal Cancer Diagnosis Using Machine Learning Based on Immunohistochemistry
Pathological Images. Sci. Rep..

[116] Kumar A., Aravind N., Gillani T., Kumar D. (2025). Artificial
Intelligence Breakthrough in Diagnosis, Treatment, and Prevention
of Colorectal Cancer – A Comprehensive Review. Biomed. Signal Process. Control.

[117] Caruso D., Polici M., Bellini D., Laghi A. (2024). ESR Essentials:
Imaging in Colorectal Cancer—Practice Recommendations by ESGAR. Eur. Radiol..

[118] Baeßler B., Maintz D., Persigehl T. (2016). Imaging Procedures
for Colorectal Cancer. Visc. Med..

[119] Moreno C., Kim D. H., Bartel T. B., Cash B. D., Chang K. J., Feig B. W., Fowler K. J., Garcia E. M., Kambadakone A. R., Lambert D. L., Levy A. D., Marin D., Peterson C. M., Scheirey C. D., Smith M. P., Weinstein S., Carucci L. R. (2018). ACR Appropriateness Criteria®
Colorectal Cancer
Screening. J. Am. Coll. Radiol..

[120] Menozzi L., Yao J. (2024). Deep Tissue Photoacoustic Imaging
with Light and Sound. Npj Imaging.

[121] Steinberg I., Huland D. M., Vermesh O., Frostig H. E., Tummers W. S., Gambhir S. S. (2019). Photoacoustic Clinical
Imaging. Photoacoustics.

[122] Sun Z., Du J. (2021). Suppression of Motion Artifacts in
Intravascular Photoacoustic
Image Sequences. Biomed. Opt. Express.

[123] Wei, J. ; Song, X. ; Wang, Q. ; Luo, Q. ; Yang, X. Prospective Respiration-Gated Optical-Resolution Photoacoustic Microscopy to Eliminate Motion Artifacts Caused by Respiratory of Mouse. In Optical Interactions with Tissue and Cells XXXII; SPIE, 2021; Vol. 11640, pp. 61–66.

[124] Zheng S., Jiejie D., Yue Y., Qi M., Huifeng S. (2023). A Deep Learning Method for Motion Artifact Correction
in Intravascular Photoacoustic Image Sequence. IEEE Trans. Med. Imaging.

[125] Kuniyil Ajith Singh M., Xia W. (2020). Portable and Affordable
Light Source-Based
Photoacoustic Tomography. Sensors.

[126] Mehrmohammadi M., Yoon S. J., Yeager D., Emelianov S. Y. (2013). Photoacoustic
Imaging for Cancer Detection and Staging. Curr.
Mol. Imaging.

[127] Liu H., Wang M., Ji F., Jiang Y., Yang M. (2024). Mini Review
of Photoacoustic Clinical Imaging: A Noninvasive Tool for Disease
Diagnosis and Treatment Evaluation. J. Biomed.
Opt..

[128] Assi H., Cao R., Castelino M., Cox B., Gilbert F. J., Gröhl J., Gurusamy K., Hacker L., Ivory A. M., Joseph J., Knieling F., Leahy M. J., Lilaj L., Manohar S., Meglinski I., Moran C., Murray A., Oraevsky A. A., Pagel M. D., Pramanik M., Raymond J., Singh M. K. A., Vogt W. C., Wang L., Yang S., Bohndiek S. E. (2023). A Review of a Strategic
Roadmapping Exercise to Advance Clinical Translation of Photoacoustic
Imaging: From Current Barriers to Future Adoption. Photoacoustics.

[129] Peng Y., Deng H. (2024). Medical Image Fusion
Based on Machine
Learning for Health Diagnosis and Monitoring of Colorectal Cancer. BMC Med. Imaging.

[130] Yang J., Choi S., Kim J., Park B., Kim C. (2023). Recent Advances
in Deep-Learning-Enhanced Photoacoustic Imaging. Adv. Photonics Nexus.

